# miR579-3p is an inhibitory modulator of neointimal hyperplasia and transcription factors c-MYB and KLF4

**DOI:** 10.1038/s41420-023-01364-7

**Published:** 2023-02-22

**Authors:** Xiujie Xie, Takuro Shirasu, Jing Li, Lian-Wang Guo, K. Craig Kent

**Affiliations:** 1 Department of Surgery, School of Medicine, University of Virginia, Charlottesville, VA 22908 USA; 2 Department of Molecular Physiology and Biological Physics, University of Virginia, Charlottesville, VA 22908 USA; 3 Robert M. Berne Cardiovascular Research Center, University of Virginia, Charlottesville, VA 22908 USA

**Keywords:** Biochemistry, Pathogenesis

## Abstract

Neointimal hyperplasia (IH) is a common vascular pathology that typically manifests in in-stent restenosis and bypass vein graft failure. Smooth muscle cell (SMC) phenotypic switching is central to IH, both regulated by some microRNAs, yet the role of miR579-3p, a scarcely studied microRNA, is not known. Unbiased bioinformatic analysis suggested that miR579-3p was repressed in human primary SMCs treated with different pro-IH cytokines. Moreover, miR579-3p was software-predicted to target both c-MYB and KLF4 − two master transcription factors known to promote SMC phenotypic switching. Interestingly, treating injured rat carotid arteries via local infusion of miR579-3p-expressing lentivirus reduced IH 14 days after injury. In cultured human SMCs, transfection with miR579-3p inhibited SMC phenotypic switching, as indicated by decreased proliferation/migration and increased SMC contractile proteins. miR579-3p transfection downregulated c-MYB and KLF4, and luciferase assays indicated miR579-3p’s targeting of the 3′UTRs of the c-MYB and KLF4 mRNAs. In vivo, immunohistochemistry showed that treatment of injured rat arteries with the miR579-3p lentivirus reduced c-MYB and KLF4 and increased SMC contractile proteins. Thus, this study identifies miR579-3p as a previously unrecognized small-RNA inhibitor of IH and SMC phenotypic switch involving its targeting of c-MYB and KLF4. Further studies on miR579-3p may provide an opportunity for translation to develop IH-mitigating new therapeutics.

## Introduction

Stenotic cardiovascular diseases, in particular, atherosclerosis, are the leading cause of morbidity and mortality worldwide despite improvements in medical therapies [1]. Currently, two major clinical approaches are used to resume obstructed blood flow: angioplasty and bypass surgery [2, 3]. Unfortunately, following either procedure, neointimal hyperplasia (IH) develops in the vessel wall, and if untreated, could narrow the vessel lumen and contribute to recurrent stenosis or bypass graft failure. Drug-eluting stents have been advanced to treat post-angioplasty IH but in-stent restenosis still occurs. Moreover, the drugs used on the stents (e.g. paclitaxel) which are indiscriminately anti-proliferative heighten risks of thrombosis and mortality, as increasingly reported [4–6]. To date, there has been a lack of approved therapeutics to prevent bypass grafts from developing IH and subsequent post-surgical graft failure [7–9]. Undoubtedly, postoperative IH, whether associated with angioplasty or bypass surgery, remains a major medical problem, and better/specific therapeutic agents for IH prevention are critically needed.

Vascular smooth muscle cells (SMCs) constitute major cellular populations and crucial functions in the normal vessel wall. However, when exposed to stimulants in a disturbed microenvironment, such as cytokines that surge in the injured vascular wall, SMCs undergo contractile-to-synthetic phenotypic switching, becoming proliferative, migratory, and de-differentiated (or further transdifferentiated to other cell types) [10, 11]. Consequently, highly cellular neointimal lesions arise, occupying luminal space. While IH is complex involving many cell types and events, recent studies applying lineage tracing and single-cell sequencing support that phenotypic changes of resident SMCs play a central role in IH-associated diseases [11–13].

A fraction of transcription factors (TFs), so-called master TFs, are critically important for maintaining or switching cell phenotypes in various sources of cells [14, 15], among which KLF4 and c-MYB have been well established as crucial for SMC phenotypic switching. While KLF4 strongly promotes SMC de-differentiation by inhibiting the expression of SMC markers (contractile proteins) in vitro and in arteries undergoing IH [16, 17], c-MYB perpetuates SMC proliferation and migration; reducing c-MYB activity hampers IH in arteries and veins in animal models [18–22]. In this light, blockade of IH-driving master TF activities would provide an effective means to mitigate IH [19]. However, TFs are not typical drug targets, and currently, small-molecule inhibitors are not available for most of TFs including c-MYB and KLF4. This underscores the importance of alternative approaches to targeting TFs.

In the past decade, microRNAs (miRs) have emerged as promising candidate therapeutics [23]. This class of small non-coding RNAs is posttranscriptional regulators closely involved in development and disease [24–28]. They negatively regulate gene expression via complementary sequences in the 3′UTR of the target gene mRNA. Some miRs have been found to be regulators of IH and have attracted considerable attention in vascular research [28–38]. In particular, the miR145 cluster and the miR200 cluster help maintain the SMC contractile phenotype [29, 35]. On the other hand, miR21 promotes SMC phenotypic switching exacerbating IH in arteries [39] and veins [38]. In a translational endeavor using a humanized model, the implantation of stents coated with anti-miR against miR21 reduced IH [39]. Despite these advances, miR-mediated molecular mechanisms and therapeutic targeting thereof are not adequately studied in the context of IH.

In our previous study [40], through unbiased analysis of microarray and bioinformatics, we established an approach to screening miRs that are responsive to each of the 4 salient cytokines that surge after vascular injury, namely, PDGF-BB, TGFβ1, TNFα, and IL1β. Among these miRs, miR579-3p is rarely studied and herein software-predicted to target not only c-MYB but also KLF4, two master transcription factors key to SMC and neointima pathophysiology. Moreover, previous studies indicated decreased miR579-3p levels in cancer cells and serum samples from patients of melanoma and hepatocellular carcinoma [41–43], consistent with an anti-proliferative function of miR579-3p. Herein, we hypothesized that miR579-3p could confer an inhibitory effect on pro-IH SMC behaviors. Interestingly, using a rat model of arterial injury, we found that treating injured arteries with lentivirus to express miR579-3p reduced IH. Our in vivo and in vitro findings suggest that miR579-3p could be used to preserve SMC phenotypic stability in combating IH.

## Results

### miR579 is a novel IH-mitigating modulator

**Fig. 1:**
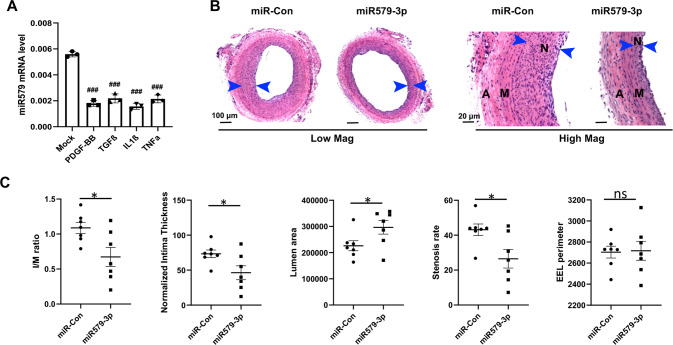
Treatment of injured rat carotid arteries with miR579-3p-expressing lentivirus inhibits injury-induced IH. **A** In vitro experiments showing down-regulation of miR579-3p in AoSMCs by a panel of pro-IH cytokines. AoSMCs were starved in basal medium (no FBS) for 24 h and then treated for 24 h without (Mock control) or with a cytokine (50 ng/ml PDGF-BB, 20 ng/mL TGFβ1, 20 ng/ml TNFα, or 10 ng/ml IL1β). miR579-3p mRNA was measured by qRT-PCR. Data are presented as mean ± SD (*n* = 3 replicates). ^###^*P* < 0.001 compared to the Mock control, as analyzed with one‐way ANOVA followed by Bonferroni post hoc test. **B**, **C** In vivo studies showing inhibition of IH by the treatment with miR579-3p-expressing lentivirus. IH was induced by balloon injury in the rat common carotid artery, and the lentivirus used to express scrambled microRNA or miR579 was locally infused into the injured artery wall. Arteries were collected at post-injury day 14 for histology and morphometric analyses. Representative H&E-stained artery cross‐sections are shown in B. A pair of arrows define the neointima thickness. A, adventitia; M, media; N, neointima. Scale bar: 100 μm (Low mag.), 20 μm (High mag). Quantitative morphometric analysis (C): IH = neointima *vs* media (I/M) area ratio, normalized neointima thickness, lumen area, stenosis rate, and EEL perimeter. The data values of 3–4 sections from each animal were averaged. The averages from seven animals in each treatment group were then averaged again to produce mean ± SEM. *p < 0.05, *n* = 7 rats, unpaired Student’s t-test.

Common vascular reconstructive procedures, whether angioplasty or bypass grafting, expose the medial SMCs to a myriad of cytokine stimuli that trigger IH-forming SMC phenotypic changes. Salient cytokines include PDGF-BB, TGFβ1, TNFα, and IL1β. PDGF-BB has been widely used to stimulate SMC phenotypic switching in cell culture. However, the microenvironment of SMCs in the damaged vascular wall is complex, and so is IH pathogenesis. Therefore, we adopted a more holistic approach in our previous microarray study [40] to screen miRNAs that robustly respond to all 4 aforementioned cytokines rather than PDGF-BB alone. Unbiased screening and bioinformatics suggested that miR579-3p would be a novel regulator of SMC plasticity because it was downregulated by either of the 4 cytokines (Fig. 1A, Fig. S2A) and interestingly, also predicted to target both KLF4 and c-MYB. There are only a very small number of reports related to miR579-3p, mainly involving cancer cells [41, 44–46]. Its function in SMCs and IH was not known.

To explore its potential role as an IH regulator, we conducted angioplasty to injure rat carotid arteries thereby inducing IH [47], and infused lentivirus locally into the damaged artery wall to express miR579-3p or miR-Con (control microRNA). Morphometric parameters were measured on cross-sections of the arteries collected 14 days after angioplasty (Fig. 1B). As indicated by the quantitative data in Fig. 1C, the treatment with the miR579-3p-expressing lentivirus (*vs* miR-Con) significantly reduced IH, whether presented as the neointima/media area ratio (I/M) or normalized neointima thickness. Accordingly, stenosis was mitigated, as indicated by significantly increased lumen area and decreased stenosis rate. There was no vessel shrinkage, an adverse outcome, as no change was observed in the overall vessel size (EEL perimeter). Thus, these results uncovered miR579-3p as a novel negative regulator of IH.

### miR579-3p is a negative regulator of SMC phenotypic switching

**Fig. 2:**
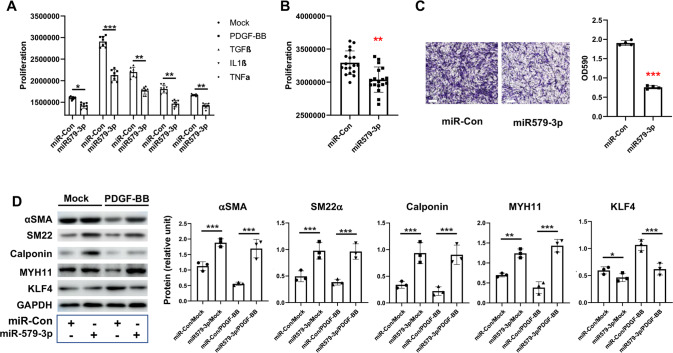
miR579-3p suppresses AoSMC proliferation, migration, and de-differentiation. **A**, **B** Proliferation assay. AoSMCs starved in basal medium (no FBS, 24 h) were transfected with miR-con or miR579-3p for 24 h. The medium was then changed to fresh basal medium with or without a cytokine (**A**) (50 ng/ml PDGF-BB, 20 ng/mL TGFβ1, 20 ng/ml TNFα, or 10 ng/ml IL1β) or full medium (**B**) for an additional 24 h before the CellTiter‐Glo viability assay. **C** Migration assay. AoSMCs were seeded in the Transwell insert, the lower chamber filled with full medium. Cells that migrated to the lower surface of the insert were imaged after a 24 h incubation. To quantify the migration, 33% (v/v) Acetic acid was added into the insert to elute the bound crystal violet, and then the eluent from the lower chamber was measured for absorbance (590 nm) using a 96-well microplate reader. Scale bar: 200 μm. **D** De-differentiation (Western blotting of SMC contractile proteins). AoSMCs were starved (no FBS, 24 h) and transfected with miR-con or miR579-3P for 24 h, and then treated with or without 50 ng/ml PDGF-BB for another 24 h. Quantification for A-D: Mean ± SD (**A** and **B**, see dot plots for the number of replicates) or mean ± SEM (**C** and **D**, *n* = 3 independent repeat experiments). Pairwise comparison was made through Student’s *t*-test. **P < 0.01, ***P < 0.001.

It has been well documented that injury-induced IH is primarily formed by SMCs that have undergone phenotypic switching [13, 48]. We thus next determined the role of miR579-3p in this process. We found that increasing miR579-3p in human primary aortic SMCs (AoSMCs) via transfection markedly reduced (*vs* miR-Con) AoSMC proliferation in the presence of either of the 4 cytokines, namely, PDGF-BB, TGFβ1, TNFα, and IL1β (Fig. 2A). This proliferation-blunting effect was also observed with AoSMCs cultured in full medium (Fig. 2B). Moreover, miR579-3p reduced the migration of AoSMCs cultured in full media by ~60% (Fig. 2C).

We then determined the effect of miR579-3p transfection on SMC de-differentiation through Western blotting of a panel of contractile protein markers. Since PDGF-BB among the 4 cytokines is most commonly used to robustly induce SMC de-differentiation, we chose PDGF-BB as the stimulant for this experiment. Interestingly, the data in Fig. 2D indicates that miR579-3p drastically increased SMC contractile proteins αSMA, SM22, calponin, and MYH11, which were reduced by PDGF-BB in the presence of miR-Con. The effect of miR579-3p on elevating SMC marker proteins was also observed in the absence of PDGF-BB albeit to a lesser extent. Of note, while PDGF-BB upregulated KLF4, the master TF that represses the expression of SMC contractile genes [17], the miR579-3p transfection of AoSMCs brought KLF4 protein back to the basal level.

Thus, these results collectively demonstrate that miR579-3p suppresses AoSMC proliferation, migration and de-differentiation, we have therefore identified a novel miR regulator of SMC phenotypic switching.

### miR579-3p negatively regulates c-MYB expression

**Fig. 3:**
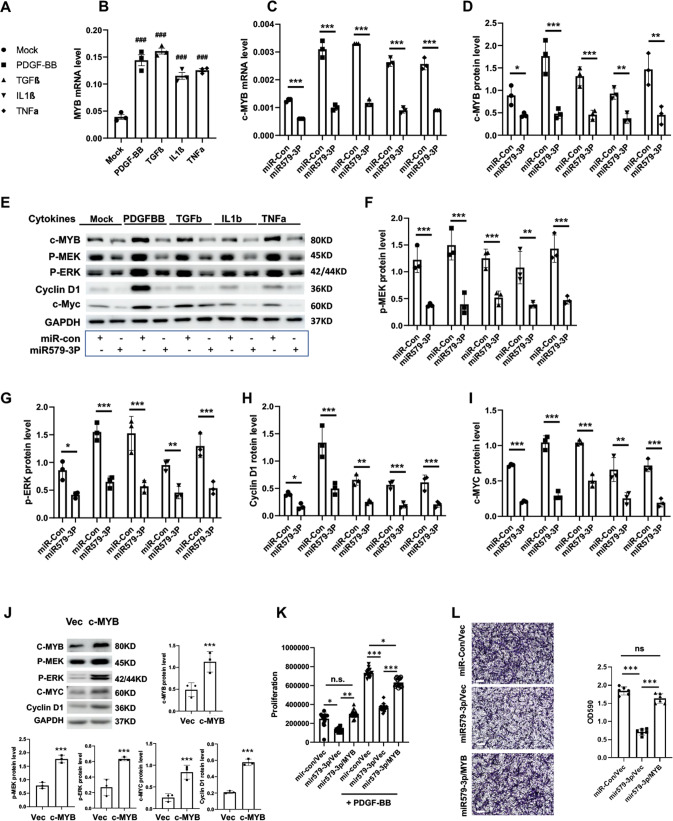
miR579-3p represses the expression of c-MYB and proliferation/migration marker proteins. AoSMCs starved in basal medium (no FBS, 24 h) were transfected with miR-con or miR579-3p for 24 h. The medium was then changed to fresh basal medium with or without a cytokine (50 ng/ml PDGF-BB, 20 ng/mL TGFβ1, 20 ng/ml TNFα, or 10 ng/ml IL1β) or full medium (migration assay) for an additional 24 h prior to various assays. **A** Lend of symbols (for **B–I**). **B** qRT-PCR showing upregulation of c-MYB mRNA by cytokines. **C** qRT-PCR showing negative regulation of c-MYB mRNA by miR579-3p. **D–I** Western blots showing that miR579-3P negatively regulates protein levels of c-MYB and proliferation/migration markers. Phospho-protein levels were normalized to the loading control GAPDH. **J** Western blots showing that c-MYB overexpression increases proliferation/migration markers. **K** Proliferation assay indicating that c-MYB overexpression rescues miR579-3P-mitigated AoSMC proliferation. PDGF-BB was included in the SMC culture. **L** Transwell assay indicating that c-MYB overexpression rescues miR579-3P-inhibited AoSMC migration. Scale bar: 200 μm. Quantification for **A–L**: Data are presented as mean ± SD (*n* = 3 replicates in **B** and **C**; *n* = 6 replicates in **K** and **L**) or mean ± SEM (western blots, *n* = 3 independent repeat experiments in **D–J**); ^###^*P* < 0.001 (compared to Mock control, the first bar), as analyzed with one‐way ANOVA followed by Bonferroni post hoc test. Pairwise comparison was made through Student’s *t*-test: *P < 0.05, **P < 0.01, ***P < 0.001.

In the pursuit of the molecular mechanisms that underlie the miR579-3p regulation of SMC phenotype, we noticed opposite trends of cytokine-induced gene expression of miR579-3p and c-MYB (Fig. S2B). Whereas miR579-3p was downregulated, c-MYB was upregulated by all 4 cytokines in AoSMCs (Fig. S2C). This inversed correlation was verified by qRT-PCR (Figs. 1A and 3B). While c-MYB is best known as a potent oncogenic factor, its role as a master TF that stokes SMC/neointimal proliferation has also been reported [19]. We thus hypothesized that miR579-3p inhibited SMC proliferation and migration by downregulating c-MYB. Indeed, whereas each of the 4 cytokines upregulated c-MYB mRNA by at least twofold, transfection of AoSMC with miR579-3p abrogated the cytokine effect (Fig. 3C). Accordingly, miR579-3p also kept c-MYB protein at the basal level (no cytokine) as indicated by Western blotting (Fig. 3D, E). Consistently, the major SMC proliferation/migration markers, including p-MEK, p-ERK, cyclin-D1, and c-MYC, were all significantly reduced by miR579-3p (Fig. 3F–I). In accordance with previous reports in other cell types showing that these markers are c-MYB downstream target genes, herein we observed that elevating c-MYB protein levels potently increased these 4 marker proteins (Fig. 3J). More importantly, we found that while miR579-3p transfection into AoSMCs reduced (*vs* moR-Con) cell proliferation and migration, overexpression of c-MYB in this miR579-3p background largely reversed the miR579-3p effect (Fig. 3K, L). Taken together, these results reveal miR579-3p as a novel negative regulator of c-MYB expression and its function in SMC proliferation and migration.

### miR579-3p targets two sequences in the c-MYB 3′UTR

**Fig. 4:**
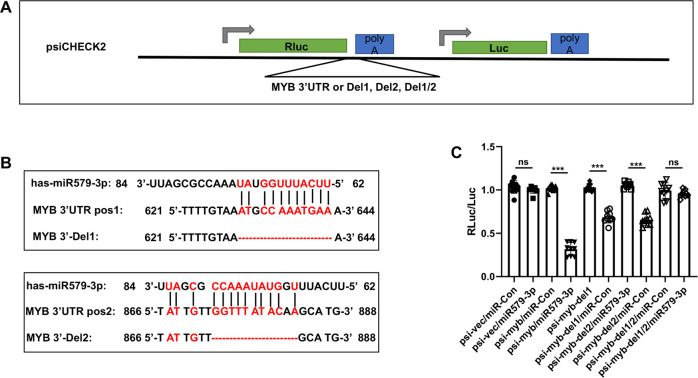
miR579-3p directly targets the c-MYB mRNA 3′UTR. The cDNA of wild-type 3′-UTR of c-MYB was cloned from the human genome. The sequences of wild type and mutants with 12 bp deletion at position 1, 11 bp deletion at position 2, or deletion of both were amplified and subcloned into the XhoI and NotI sites between Renilla gene and polyA of the psiCheck2 vector. Each constructed luciferase reporter plasmid or empty vector was transfected into AoSMCs (5000 cells per well) in 96-well plates. Luciferase was assayed at 48 h post transfection using the Dual-Luciferase Reporter Assay System. Luminescence was quantitated and renilla luciferase readings were normalized against the firefly luciferase activity to determine the relative luciferase activity. Data are presented as mean ± SD (*n* = 8 replicates). Pairwise comparison was made through Student’s *t*-test: ***P < 0.001; n.s. not significant. **A** Schematic of the luciferase reporter vector and constructs. **B** The miR579-3p sequences complementary to that at positions 1 and 2 of c-MYB 3′UTR. **C** Relative luciferase activity compared between miR579-3p and miR-Con.

The negative regulation of c-MYB expression by miR579-3p led us to infer that c-MYB could be a direct target of miR579-3p. Indeed, via in-silico analysis, we found two positions in the 3′UTR of c-MYB mRNA that are complementary to the sequence of mature miR579-3p. We then used luciferase activity for readout to determine how deletion of these sequences could affect miR579-3p’s targeting of c-MYB mRNA. The wild-type 3′UTR of c-MYB, 3′UTR with position 1 deleted, 3′UTR with position 2 deleted, or 3′UTR with both deletions was inserted into the downstream of the Renilla gene (Fig. 4A). The sequences of position 1 and position 2 in c-MYB 3′UTR and complementary sequences in miR579-3p are presented in Fig. 4B. As indicated in Fig. 4C, luciferase activity was remarkably reduced by miR579-3p treatment when the wild 3′UTR construct was used. However, deletion of either position1 or position 2 partially restored, and deletion of both completely restored the luciferase activity. Thus, the luciferase assay data demonstrate that c-MYB is a direct target of miR579-3p.

### miR579-3p targets a sequence in the KLF4 3′UTR

**Fig. 5:**
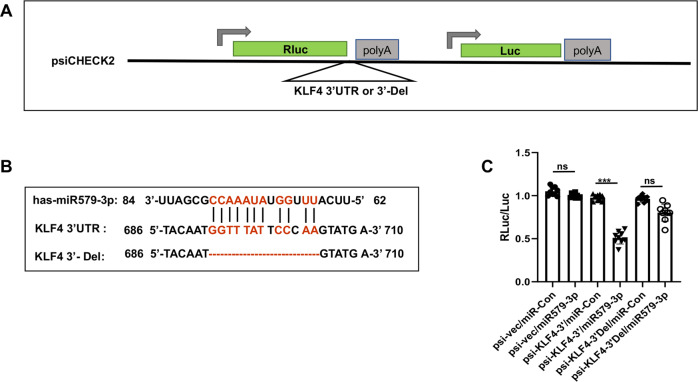
miR579-3p targets the KLF4 mRNA 3′UTR. Wild-type and 13 bp deletion of 3′-UTR of KLF4, was amplified from the human genome and subcloned into the XhoI and NotI sites between Renilla gene and polyA of the psiCheck2 vector. Each constructed luciferase reporter plasmid or empty vector was transfected into AoSMCs at 5000 cells per well in 96-well plates. Luciferase was assayed at post 48 h transfection using the Dual-Luciferase Reporter Assay System. Luminescence was quantitated and renilla luciferase readings were normalized against the firefly luciferase activity to determine the relative luciferase activity. Data are presented as mean ± SD (*n* = 8 replicates). Pairwise comparison was made through Student’s t-test: ***P < 0.001; n.s. not significant. **A** Schematic of the luciferase reporter vector and constructs. **B** The miR579-3p sequence complementary to that of the KLF4 3′UTR. **C** Relative luciferase activity compared between miR579-3p and miR-Con.

Furthermore, given that KLF4 expression was negatively regulated by miR579 (Fig. 2D) and in-silico analysis predicted a potential miR579’s target site in the KLF4 mRNA sequence, we examined whether KLF4 could be another target of miR579-3p. We performed a luciferase assay, using the psiCHECK vector inserted with the wild-type 3′UTR of KLF4 or the 3′UTR with a deletion of the 13 bp predicted miR579-3p target sequence (Fig. 5A). The deleted c-MYB 3′UTR sequence and the complementary sequence of miR579-3p are presented in Fig. 5B. We found that while the luciferase activity resulting from the wild 3′UTR construct was reduced by miR579-3p treatment of AoSMCs, it was fully restored by the deletion of the predicted target sequence.

Taken together, the luciferase assays confirm that miR579-3p directly targets c-MYB and KLF4, two master TFs dictating SMC proliferation/migration and de-differentiation [16], in accordance with down-regulation of c-MYB and KLF4 and mitigation of AoSMC phenotypic switching caused by the treatment with miR579-3p, as observed herein in vitro.

### c-MYB and KLF4 are downregulated in injured rat aortic arteries treated with miR579-3p-expressing lentivirus

**Fig. 6:**
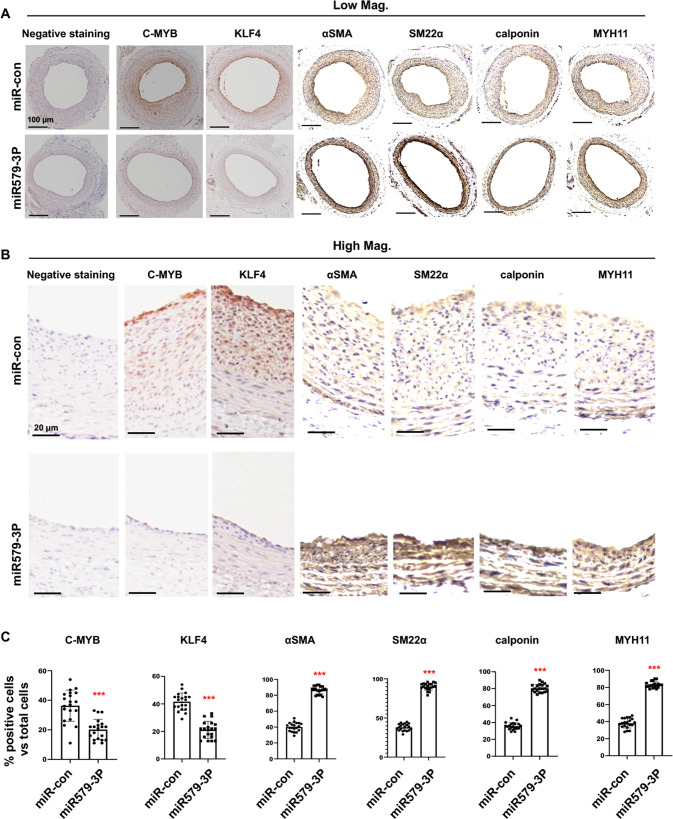
Treatment of injured rat carotid arteries with the miR579-3p lentivirus reduces c-MYB and KLF4 expression and increases SMC contractile proteins. The lentivirus for expressing miR579-3p or miR-Con was infused into the rat carotid artery wall immediately after balloon injury. The arteries were collected 14 days after injury and immunohistochemistry was performed on cross-sections to detect c-MYB, KLF4, αSMA, SM22, Calponin, and MYH11. **A**, **B** Representative immunostained cross‐sections (low-mag and high-mag, respectively). Negative staining: no primary antibody. Scale bar: 100 μm for low-mag pictures and 20 μm for high-mag pictures. **C** Quantification. Three sections were used for each animal, and the data values (colorimetric intensity per image field) from total 7 rats in a treatment group were averaged; mean ± SD (*n* = 20 or 21 sections). Statistics: Unpaired Student’s *t*-test; *P < 0.05, **P < 0.01, ***P < 0.001.

After identifying the two master TFs as novel targets of miR579-3p in AoSMCs in vitro, we next performed immunohistochemistry to confirm this novel miR579-3p-mediated regulation in vivo using cross-sections of injured arteries, which were transduced with lentivirus to express miR579-3p or miR-Con (Fig. 6A, B). Consistent with the in vitro results, treatment of injured arteries with miR579-3p-expressing lentivirus markedly reduced not only c-MYB, but also KLF4 (Fig. 6C). Accordingly, the contractile proteins which are known to be negatively regulated by KLF4 [16] were all increased at least two-fold. Thus, these in vivo results further support the in vitro finding that miR579-3p targets the two master TFs, which are critical players in SMC phenotypic switching and the development of IH [17].

## Discussion

IH develops after reconstruction (e.g. angioplasty, bypass) of vascular vessels, leading to recurrent stenosis − a persistent problem despite advances in medical technologies [5]. Recently, miRs have shown promise for specific targeting of IH pathogenesis. Some miRs have been reported to regulate IH [19, 35, 49, 50], with several being inhibitors of SMC phenotypic switching [35, 36, 51–53]. The miR145 cluster is in this IH-inhibitor category [29]. However, its physiological abundance is high, limiting the therapeutic potential of further increasing its levels. miR128-3p, however, is expressed at lower levels, and is inhibitory to SMC phenotypic switching and IH [36]. Thus, previous studies encourage further investigation into miRs as potential IH-inhibitory therapeutics.

Here we found that miR579-3p is a novel IH-inhibiting modulator. Consistently, transfecting human SMCs with miR579-3p abrogated mitogen-stimulated SMC phenotypic switching, the principal contributor to IH development [54, 55]. We also found that miR579-3p directly targeted c-MYB and KLF4, two master TFs critically involved in SMC phenotypic switching. Thus far, there are only a very small number of reports involving miR579-3p, mainly in the oncology field. While the expression of miR579-3p was suppressed in several malignant carcinomas, miR579-3p was found to inhibit cancer cell proliferation and migration [41, 44–46]. This is in line with our result that miR579-3p inhibits human SMC proliferation and migration.

Through in-silico analysis, we detected 3′UTR sequences in KLF4 and c-MYB genes that are complementary to the miR579-3p sequence. Indeed, in luciferase assays, deletion of these sequences functionally confirmed their targeting by miR579-3p. KLF4 is known as a master TF that represses the expression of SMC contractile genes [12, 16]. In accordance, our data showed that transfection of SMCs with miR579-3p in vitro or lentiviral transduction for its expression in vivo reduced KLF4 protein and increased SMC contractile proteins. It is interesting to note that in a previous report, SMC-specific KLF4 depletion in mice did not reduce but rather increased IH [17]. The exact mechanism was unclear, possibly involving an anti-proliferative role of KLF4 [17]. This outcome from KLF4 depletion underscores that targeting a single pathway would not bring about therapeutic benefits. In this regard, miRs as candidate therapeutics are advantageous for their capacity to target multiple genes or pathways [19] and could thereby assume the desired potency. Our data indicated that miR579-3p targeted both KLF4 and c-MYB. c-MYB is another master TF that potently promotes SMC proliferation and migration [20]. As a nodal factor, c-MYB governs the expression of an array of proliferation/migration markers including MEK, ERK, c-MYC, and cyclin-D1 [19]. However, c-MYB positively regulates SMC contractile genes [56–58] − a function opposite to that of KLF4 [16]. As such, these two master TFs appear to be complementary determinants in SMC phenotypic switching. In this perspective, it would be reasonable to speculate that the decrease of SMC proliferation/migration and increase of SMC contractile proteins observed after miR579-3p transfection represent the net outcome from the down-regulation of both c-MYB and KLF4. However, we cannot rule out the possibility that KLF4 may directly antagonize c-MYB’s function in the activation of SMC contractile genes. In previous studies, negating c-MYB reduced injury-induced IH [18, 20], whereas SMC-specific deletion of KLF4 increased IH [17]. It is interesting to note that in our study, expressing miR579-3p in injured rat arteries reduced both c-MYB and KLF4 and attenuated IH. One explanation would be that the observed mitigation of IH was the sum of the effects of reduced c-MYB and KLF4. Alternatively, the IH-mitigating effect of miR579-3p may be partially accounted for by additional targets of miR579-3p.

There are limitations in the current study that require future in-depth research to resolve. For example, with limited knowledge available about miR579-3p, it remains to be determined how the miR579-3p level is regulated or what are its other targets in addition to c-MYB and KLF4 in the context of IH. To definitively confirm the genes of c-MYB and KLF4 as direct targets of miR579-3p in luciferase assays, mutations should be introduced in their 3′UTR sequences where miR579-3p binds. To further delineate the function of miR579-3p in IH mitigation, SMC-specific miR579-3p knockout should be performed in mice to determine the impact on IH. Moreover, further studies are needed to elucidate the role of miR579-3p in other cell types in the vascular wall, especially given that c-MYB regulates myocardin expression in SMC’s Sca1(+) progenitor cell proliferation and differentiation [58].

## Conclusions

In the current study, we found an IH-mitigating effect of miR579-3p via lentiviral transduction of injured arteries. Moreover, transfection of cultured human SMCs with miR579-3p hampered a spectrum of IH-promoting aberrant SMC behaviors − not only proliferation and migration but also de-differentiation. This novel role of miR579-3p involves its targeting of c-MYB and KLF4, two master TFs in SMC pathobiology. Although TFs such as c-MYB and KLF4 are potential interventional targets because of their importance in diseases, it has been challenging to develop small-molecule drugs for selectively targeting these soluble proteins. To circumvent this translational barrier, miRs could be used to tune down the expression of over-active TFs. miRs are small, easy to produce, and amenable to modifications to enhance their properties, such as stability. Moreover, various delivery platforms have been developed, including nanoparticles and liposomes [59]. Therefore, miR579-3p as an inhibitory modulator of aberrant SMC phenotype and IH deserves further mechanistic and translational research.

## Materials and methods

### Materials

Human Aortic smooth muscle cells (AoSMCs, CC-2571), smooth muscle cell basal medium (SmBM, CC-3181), and SmBM plus SingleQuots of supplements (CC-3182) were purchased from Lonza. Dulbecco’s modified Eagle’s medium (DMEM, 11965118) was from Invitrogen. Lenti-X™ 293T cell line was from Clontech (632180). Recombinant Human TGF-beta 1 (240-B), TNF-alpha (210-TA), IL1 beta (201-LB), and PDGF-BB (520-BB) were from R&D Systems. Cell Titer-Glo 2.0 Assay kit (G9242), psiCHECK2 vector (C8021), and Dual-Luciferase Reporter Assay System (E1910) were from Promega. The following products were from Thermo Fisher Scientific: Scrambled microRNA control (AM4635), hsa-miR579-3p (Assay ID: MC12340), Opti‐MEM I Reduced Serum Medium (31985062), Lipofectamine RNAiMAX Transfection Reagent (13778150), TaqMan MicroRNA reverse transcription kit (4366596), TaqMan Universal Master Mix II (4440043), TaqMan primers (hsa-miR579, Assay ID: 002398; RNU44, Assay ID:001094), High-Capacity cDNA Reverse Transcription kit (4368814), TaqMan MicroRNA reverse transcription kit (4366596), PowerUp SYBR Green Master Mix (A25778), and TaqMan Universal Master Mix II (4440043). For ImmPRESS™ HRP Anti-Rabbit IgG (Peroxidase) Polymer Detection Kit (MP-7451-15) and ImmPACT DAB Peroxidase (HRP), Substrate (SK-4105) were purchased from Vector Laboratories. Transwell (12-mm diameter, 3.0 μm pore size) Polycarbonate Membrane Insert was from Corning (3402). pLenti-III-mir Control Vector (m003) and pLenti-mir579-3p (mh10891) were from Applied Biological Materials Inc. c-MYB Lentiviral Particles were from Santa Cruz (sc-400752-LAC). QuikChange II XL Site-Directed Mutagenesis Kit was purchased from Agilent (200521).

### Vascular smooth muscle cell culture

Cell cultures were maintained at 5% CO_2_ and 37 °C in a humidified incubator. AoSMCs were cultured in SmBM with aforementioned supplements. In every 3–4 days, the cells were passaged at 1:4 ratio. All the experiments were performed using AoSMCs below passage 8.

### Quantitative real-time PCR (qRT-PCR)

Total RNA was extracted from cell lysates using the TRIzol reagent. cDNA was produced using the High-Capacity cDNA Reverse Transcription kit (4368814) or the TaqMan MicroRNA reverse transcription kit (4366596). In each 20 μl reaction, 10 ng of cDNA was amplified through quantitative real-time PCR using PowerUp SYBR Green Master Mix (A25778) or TaqMan Universal Master Mix II (4440043). mRNA expression was determined using the Applied Biosystems 7900HT Fast Real-Time PCR System with validated qPCR or Taqman primers (ThermoFisher Scientific). Data were quantified using the ∆∆Ct method. The miR579 mRNA level was normalized to RNU44; other mRNAs were normalized to glyceraldehyde 3-phosphate dehydrogenase (GAPDH). qRT‐PCR was performed in triplicate reactions. qRT-PCR primers are listed in Table S1.

### Western blotting

Cells were lysed in RIPA buffer containing Halt™ Protease and Phosphatase Inhibitor Cocktail (Thermo Fisher Scientific, 78440). For protein concentration determination, the Pierce BCA Protein Assay kit (Thermo Fisher Scientific, 23227) was used. Whole-cell lysates were mixed with Laemmli loading buffer, boiled, separated by 12% SDS–PAGE, and transferred to a PVDF membrane. Subsequently, immunoblot analyses were performed using specific antibodies (see source companies, catalog numbers, and dilution ratios listed in Table S2). The specific protein bands were visualized by using Western Blotting Kit (Pierce, 35050). All the original blots are included in a supplemental file.

### Luciferase reporter construction and assay

For the c-MYB 3′UTR construct, the 1.2 kb 3′-UTR of c-MYB amplified from the human genome was subcloned into the psiCheck2 vector (Promega, C8021). QuikChange II XL Site-Directed Mutagenesis Kit (Agilent, 200521) was then used for single deletion at position 1 (629-643) or position 2 (873-885) and double deletion at both positions. In a similar fashion, the 900 bp 3′-UTR of KLF4 was amplified and subcloned into the psiCheck2 vector between the XhoI and NotI sites. In this construct, the 692-704 deletion was generated with QuikChange II XL Site-Directed Mutagenesis Kit. The primers for cloning are presented in Table S3. The clones of luciferase assay plasmids were verified for correct sequences, and then used to transfect cells in the presence of lipofectamine 3000. The set of c-MYB 3′UTR: Empty vector, wild-type 3′UTR, single deletion1, single deletion 2, or double deletions plasmid. The set of KLF4 3′UTR: Empty vector, wild-type 3′UTR, or deletion plasmid. For transfection, 5000 AoSMCs were seeded in each well of 96-well plates and cultured for 24 h in full medium, then the medium was changed to basal medium 2 h before transfection. Luciferase assay was performed at 48 h after transfection by following the manufacturer’s instruction and using Dual-Luciferase Reporter Assay System (Promega, E1910). Luminescence was quantified, renilla luciferase readings were normalized against the firefly luciferase activity, and relative luciferase activity was thereby determined.

### Treatment of AoSMCs with miR579-3p and/or cytokines

AoSMCs were cultured in the full medium until ~90% confluency, changed to the basal medium without fetal bovine serum (FBS) for 2 h, and then transfected with hsa-miR579-3p or scrambled control using Lipofectamine RNAiMAX Transfection Reagent for 12 h. The culture was then incubated with fresh basal medium (no Lipofectamine) for another 12 h. At the end of this incubation, PDGF-BB (50 ng/ml), TGFβ1 (20 ng/mL), TNFα (20 ng/ml), or IL1β (10 ng/ml) was added, and cells were harvested for assays 24 h after the cytokine treatment.

### c-MYB overexpression in AoSMCs

AoSMCs were cultured in the full medium until ~70% confluency and changed to the basal medium (0% FBS) for 2 h. The cells were infected with c-MYB-overexpressing lentivirus (sc-400752-LAC) at MOI 10 for 12 h, and changed to either full medium or basal medium. After 48 h, the cells in the full medium were collected for Western blot assay, and the cells in the basal medium were transfected with miR579-3p for 12 h prior to a 24 h cytokine treatment.

### Cell Titer-Glo viability assay

AoSMCs of equal number were seeded in each well of the 96-well plate. Cells were treated under conditions specified in figure legends. Cells were washed with PBS once, and then 50 μl of PBS, followed by adding 50 μl of the CellTiter-Glo reagent. The 96-well plate was then analyzed for cell proliferation using BioTek Gene 5 Microplate Reader (BioTek Instruments, Inc).

### Transwell migration assay

AoSMCs were grown in 12-well transwell upper inserts until 80–90% confluence. After 24 h of starvation in basal medium, cells were transfected with hsa-miR579-3p or scrambled control miR, or infected with c-MYB Lentiviral particles. Full growth medium was added to the lower chamber. After 24 h of incubation (cell migration), the inserts were washed three times with PBS. The cells on the inside of the Transwell were removed using cotton swabs. The cells on the lower surface of the membrane were stained with 0.1% crystal violet (10% methanol) for 20 min. The insert was washed three times with PBS to remove unbound crystal violet and then air-dried. The cells that migrated were imaged under a microscope, and 400 μl 33% (v/v) acetic acid was added to the insert and shaken for 10 min to elute the bound crystal violet. The eluent from the lower chamber was measured for absorbance (590 nm) using a 96-well microplate reader.

### Animals

Male Sprague-Dawley rats were purchased from Charles River Laboratories (Wilmington, MA). All animal experiments were carried out by the recommendations in the Guide for the Care and Use of Laboratory Animals of the National Institutes of Health. The protocol was approved by the Institutional Animal Care and Use Committee (IACUC) of University of Virginia. Animals were euthanized in a chamber gradually filled with CO_2_.

### Rat carotid artery balloon injury model

Rat carotid artery balloon injury was performed as we previously described [60] with minor modifications. Briefly, after induction of general anesthesia with isoflurane, a 2-French balloon catheter (Edwards Lifesciences Corp., Irvine, CA) was inserted from the left external carotid artery into the common carotid artery of male Sprague‐Dawley rats (300∼350 g), inflated at a pressure of 1.5 atm, and retracted to the distal bifurcation. This action was repeated three times, and the catheter was rotated while being retracted at the 4th time. Immediately following the surgery, scrambled or miR579-3p lentivirus were filled in the isolated common carotid artery for 25 min. This local delivery of lentivirus for gene transfer to the injured artery wall has proven effective in our previous reports [61, 62], and the miR579-3p sequence was herein confirmed to be functionally effective for rat cells via miR579-3p transfection of cultured rat primary SMCs (Fig. S1). The external carotid artery was ligated, and the blood flow was resumed. The neck incision was then suture-closed and sanitized, and the animal was left on the warm pad to recover. Isoflurane general anesthesia was applied during the surgery (through inhaling, flow rate 2 L/minute). Bupivacaine (up to 8 mg/kg) was locally injected at the incision site, and carprofen (5 mg/kg) and buprenorphine (0.05 mg/kg) were subcutaneously injected after the surgery. The rats were euthanized 14 days after surgery and common carotid arteries were collected after perfusion fixation with 4% paraformaldehyde (PFA).

### Morphometric analysis of neointimal hyperplasia (IH)

Paraffin-embedded arteries were cut into 5-μm sections and hematoxylin‐eosin (H&E) stained. Lumen area, area inside internal elastic lamina (IEL), area inside external elastic lamina (EEL), and IEL and EEL perimeters were measured on the sections using ImageJ. Calculations were conducted following previous publication [63]: Neointimal area = IEL area - lumen area; normalized intimal thickness = neointima area/IEL perimeter; stenosis rate = (neointima area/IEL area)*100; neointima/media ratio (I/M ratio) = neointima area/(area inside adventitia – IEL area). The data was generated by averaging 3–4 artery sections from each animal, and then the means from seven animals in each treatment group were averaged to get mean ± SEM (*n* = 7 rats). The morphometric parameters were measured by a student in a blinded fashion.

### Immunohistochemistry

IHC was performed as we previously described [47]. In brief, paraffin-embedded arteries were cut into 5-μm sections. Slides were deparaffinized and rehydrated through xylenes and graded alcohol series. Citrate buffer was used for antigen retrieval in a high-pressure cocker (2 h at 80 °C). Endogenous peroxidase was blocked via incubation with 3% H_2_O_2_ for 15 min. ImmPRESS™ HRP Anti-Rabbit IgG (Peroxidase) Polymer Detection Kit (Vector Laboratories, MP-7451-15) and primary antibodies were used for immunostaining of c-MYB, KLF4, αSMA, SM22, Calponin, and MYH11, which was visualized using ImmPACT DAB Peroxidase (HRP) Substrate. Six fields on each section were imaged.

### Statistical analysis

We used Prism 8.0 (GraphPad Software) for the analyses. Comparisons between experimental groups were analyzed by one-way ANOVA (for multiple groups) or Student’s *t*-test (for two groups), as specified in figure legends. Data are presented as mean ± SEM derived from independent repeat experiments or mean ± SD derived from replicates. *P* values < 0.05 were considered statistically significant.

## Supplementary information

Supplemental

Original Data File

## Data Availability

The data that support the findings of this study are available from the corresponding authors upon reasonable request. Some data may not be made available because of privacy or ethical restrictions.
