# Supplementary material for: miR579-3p is an inhibitory modulator of neointimal hyperplasia and transcription factors c-MYB and KLF4
Source: Cell Death Discov. 2023 Feb 22;9:73. doi: 10.1038/s41420-023-01364-7 (PMC9946956; doi:10.1038/s41420-023-01364-7)
Supplement: Supplementary file 1 — Supplemental [file 41420_2023_1364_MOESM1_ESM.docx]

**Supplemental Figures**

**
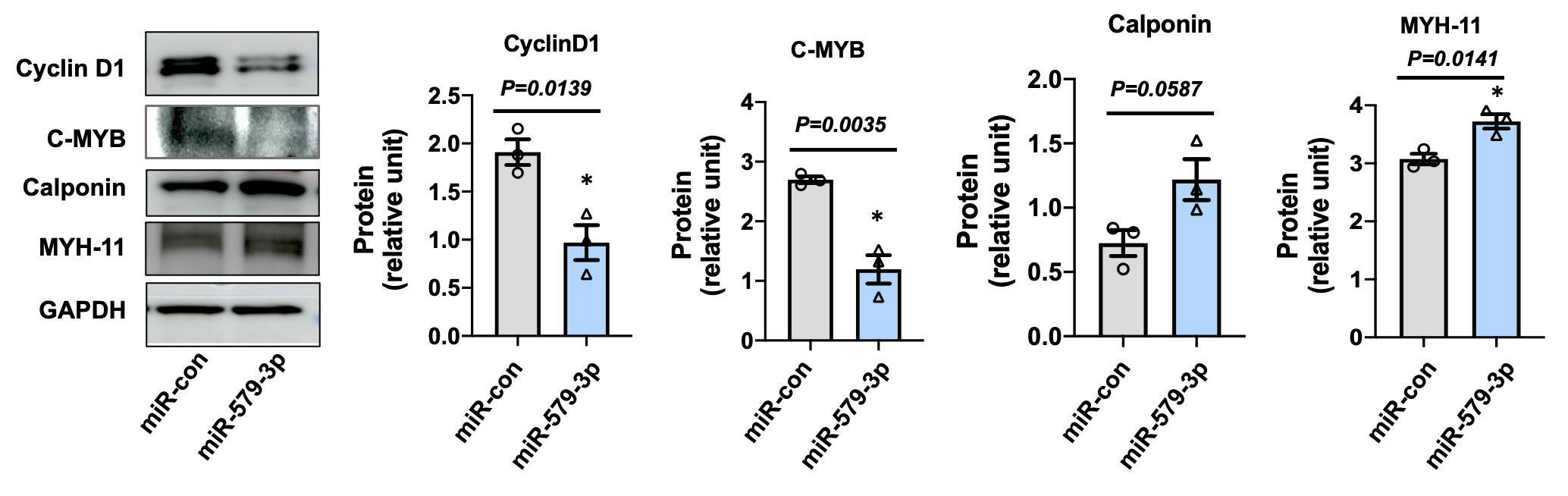
**

**Figure S1. miR579-3p represses the expression of c-MYB in rat primary SMCs**

Primary SMCs isolated from rat aortas were starved in basal medium (no FBS) for 24h, and transfected with miR-con or miR579-3p (human sequence) for 24h before Western blot assay. The Western blots show that miR579-3P negatively regulates protein levels of c-MYB and cyclin D1 and positively regulates SMC contractile proteins calponin and MYH-11.

Quantification: Data are presented as mean ± SEM, n = 3 independent repeat experiments. Student’s t-test: *P<0.05.

**Figure S2. miR579 and c-MYB gene are inversely correlated under cytokines stimulation**

Microarray was performed as described in the Methods section. AoSMCs were starved in basal medium (no FBS) for 24 h and then treated for 24h without (Mock control) or with a cytokine (50ng/ml PDGF-BB, 20 ng/mL TGFβ1, 20ng/ml TNFα, or 10ng/ml IL1β) prior to the microarray experiment.

A and B. mir579 and c-MYB expression from microarray analysis without (Mock control) or with stimulation by a cytokine (PDGF-BB, TGFβ, IL1β, or TNFα).

C. Fold change of the expression (cytokine stimulated versus Mock) of miR579 and c-MYB. The data from A and B was replotted to show an inversed relation of the changes in miR579 and c-MYB expression stimulated by each cytokine. Mean ± SD (n= 3 replicates).

**Supplemental tables**

**Table S1. Primers used for qRT-PCR**

| Target | Forward | Reverse |
| --- | --- | --- |
| **Gene expression** | | |
| c-MYB | GGGAACAGATGGGCAGAAATCG | GCTGGCTTTTGAAGACTCCTGC |
| GAPDH | CATGTTCGTCATGGGTGTGAACCA | ATGGCATGGACTGTGGTCATGAGT |

**Table S2. Antibodies for Western blotting**

| Antibody | Company | Catalog number | Dilution |
| --- | --- | --- | --- |
| c-MYB | Santa Cruz | sc-74512 | WB (1:500); IHC (1:100) |
| Phospho-MEK | Cell signaling technology | 9121 | WB (1:1000) |
| Phospho-ERK | Cell signaling technology | 4370 | WB (1:2000) |
| Cyclin D1 | Cell signaling technology | 55506 | WB (1:1000) |
| c-Myc | Cell signaling technology | 13987 | WB (1:1000) |
| GAPDH | Cell signaling technology | 2118 | WB (1:3000) |
| KLF4 | Proteintech | 11880-1-AP | WB(1:1000); IHC(1:200);IF(1:100) |
| SMA | Proteintech | 14395-1-AP | WB(1:5000); IHC(1:3000);IF(1:300) |
| SM22 | Proteintech | 10493-1-AP | WB(1:1000); IHC(1:100);IF(1:200) |
| Calponin | Proteintech | 13938-1-AP | WB(1:1000); IHC(1:100);IF(1:100) |
| MYH11 | Proteintech | 21404-1-AP | WB(1:2000); IHC(1:2000);IF(1:200) |

**Table S3. Primers for construction of c-MYB 3’UTR and KLF4 3’UTR in psiCHECK-2 vector.**

| Cloned region | Forward | Reverse |
| --- | --- | --- |
| c-MYB 3’UTR  Wild type | 5’-GACTCATACAGCTGGCACTCGAGGGACGC  TGGTCATGTGAGAC-3’ | 5’-GACTCATACAGCGGCCGCAAAGTGCC  TTGAGTCTGCTCC-3’ |
| c-MYB 3’UTR with Del1 | 5’-TTTTATTCAGTAATTTAATTTTGTAAAAACG  TTTTTTGCTGCTATGGTCTTA-3’ | 5’-TAAGACCATAGCAGCAAAAAACGTTTT  TACAAAATTAAATTACTGAATAAAA-3’ |
| c-MYB 3’UTR with Del2 | 5’-TATTGTGGTTTTTTTGTTATTGTTGCATGC  GTTGCACTTCTTTTTTGG-3’ | 5’-CCAAAAAAGAAGTGCAACGCATGCAAC  AATAACAAAAAACCACAATA-3’ |
| KLF4 3’UTR  Wild type | 5’-  ACTCATACAGCTGGCACTCGAGATCCCA  GACAGTGGATATGAC-3’ | 5’-  ACTCATACAGCGGCCGCATTCTCAC  CTTGAGAATGCA-3’ |
| KLF4 3’UTR with Del | 5’-  CAGATGTGCAATAATTTGTACAATGTA  TGCCTTAAGCAGAACAAATGTG-3’ | 5’-  CACATTTGTTCTGCTTAAGGCATA  CATTGTACAAATTATTGCACATCTG-3’ |
