# Supplementary material for: miR579-3p is an inhibitory modulator of neointimal hyperplasia and transcription factors c-MYB and KLF4
Source: Cell Death Discov. 2023 Feb 22;9:73. doi: 10.1038/s41420-023-01364-7 (PMC9946956; doi:10.1038/s41420-023-01364-7)

Figure 2D

aSMA

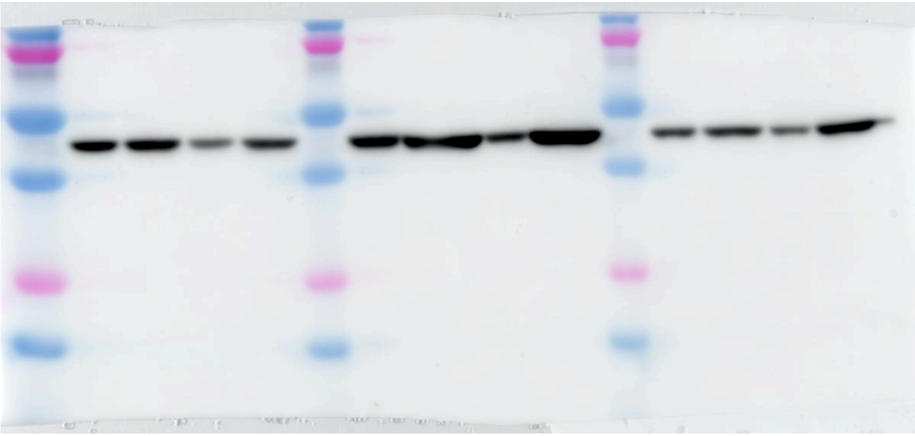

SM22a

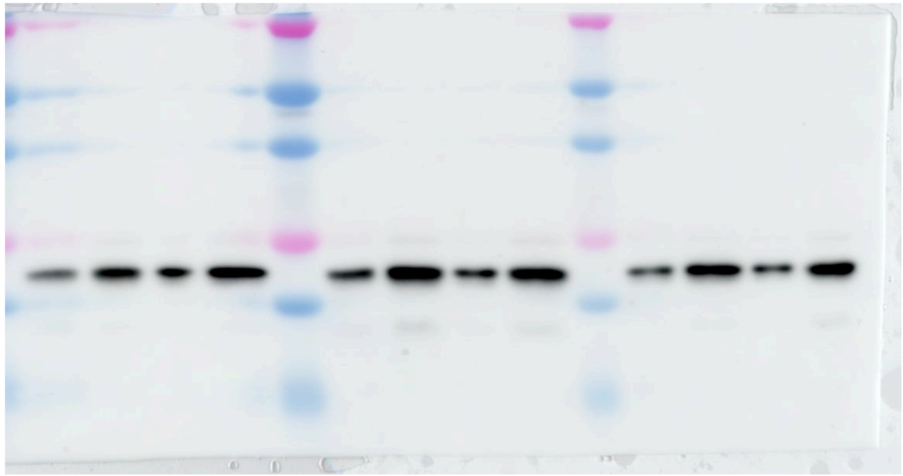

Calponin

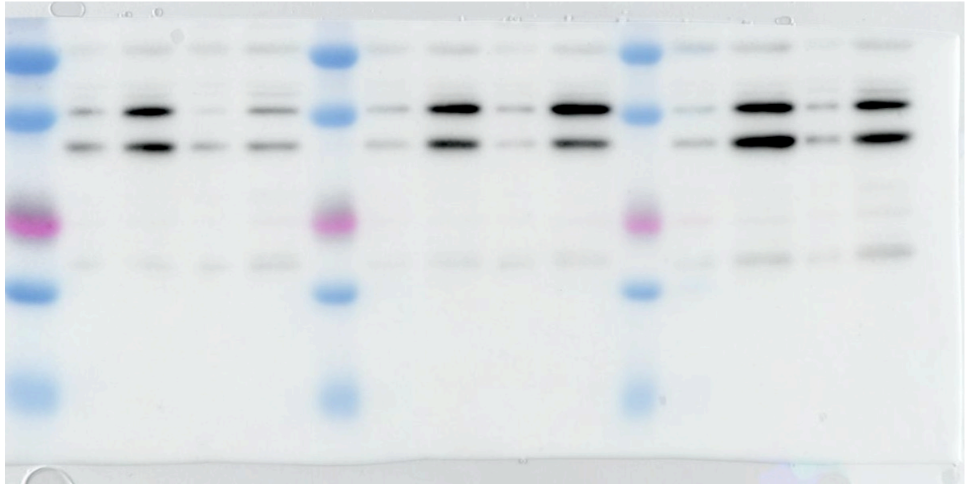

MYH11

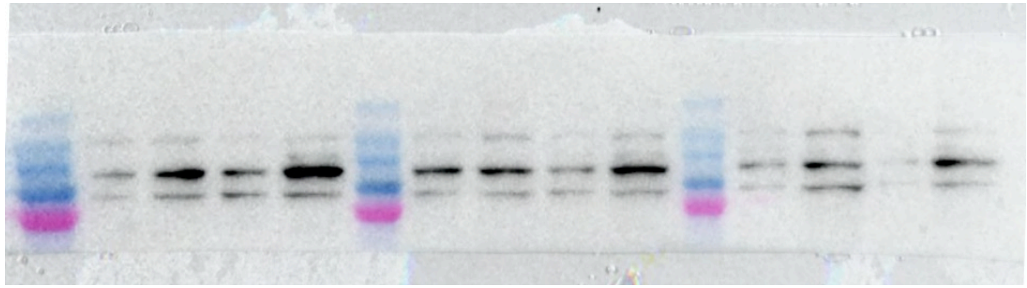

Figure 2D

KLF4

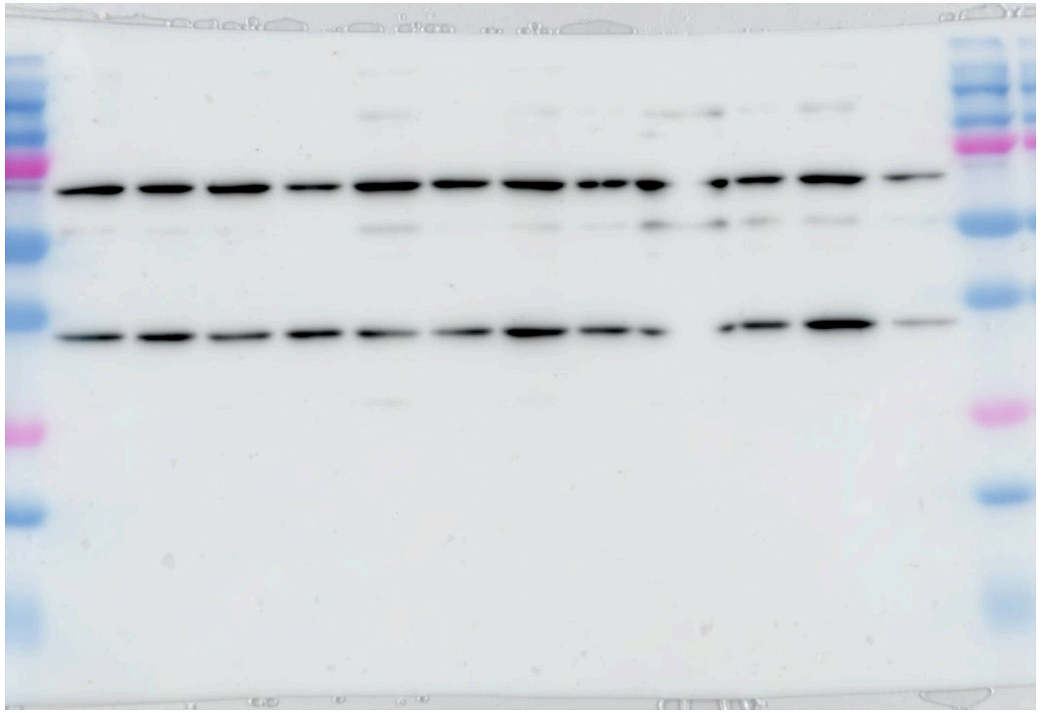

GAPDH

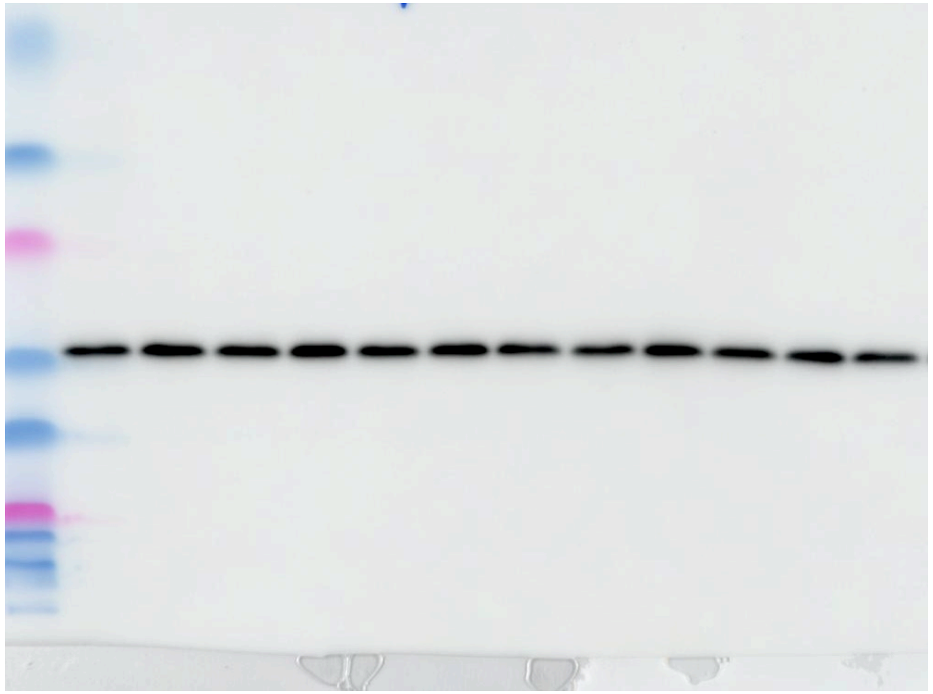

Figure 3E

C-MYB

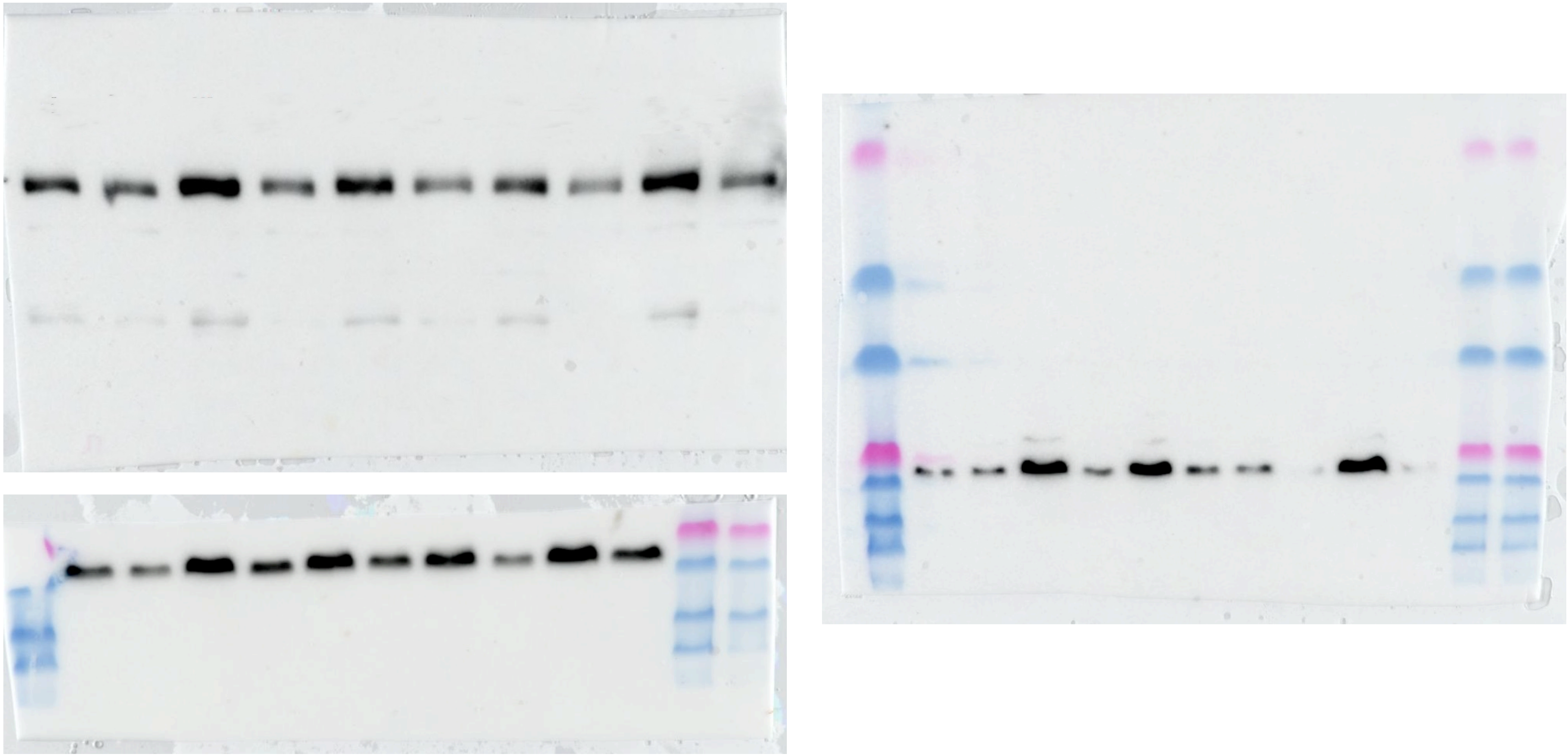

Figure 3E

P-MEK

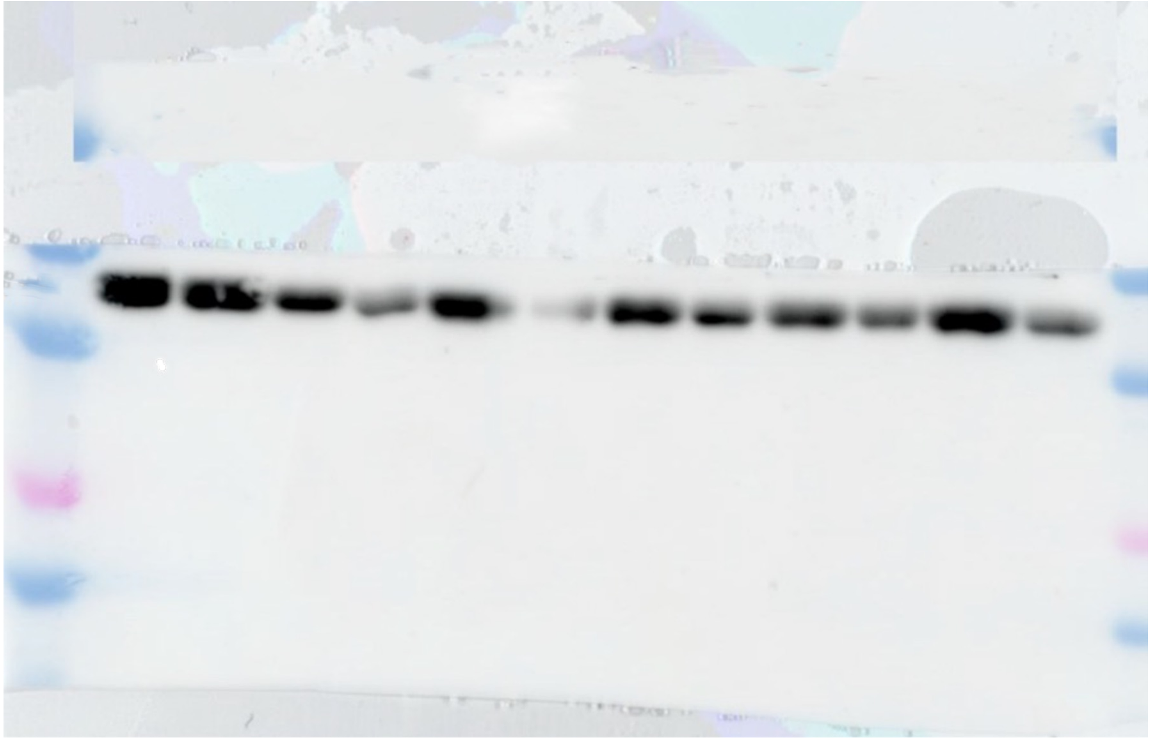

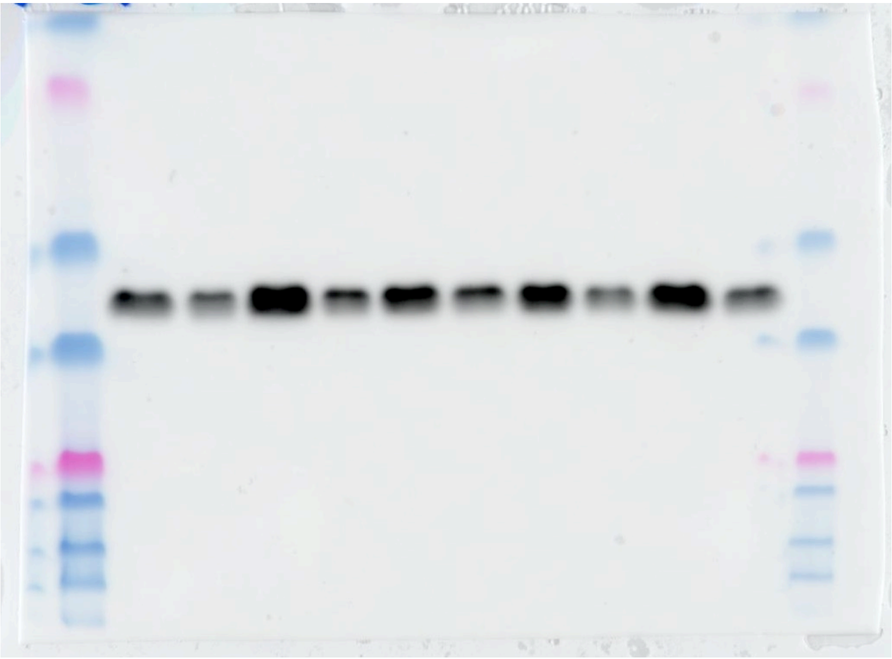

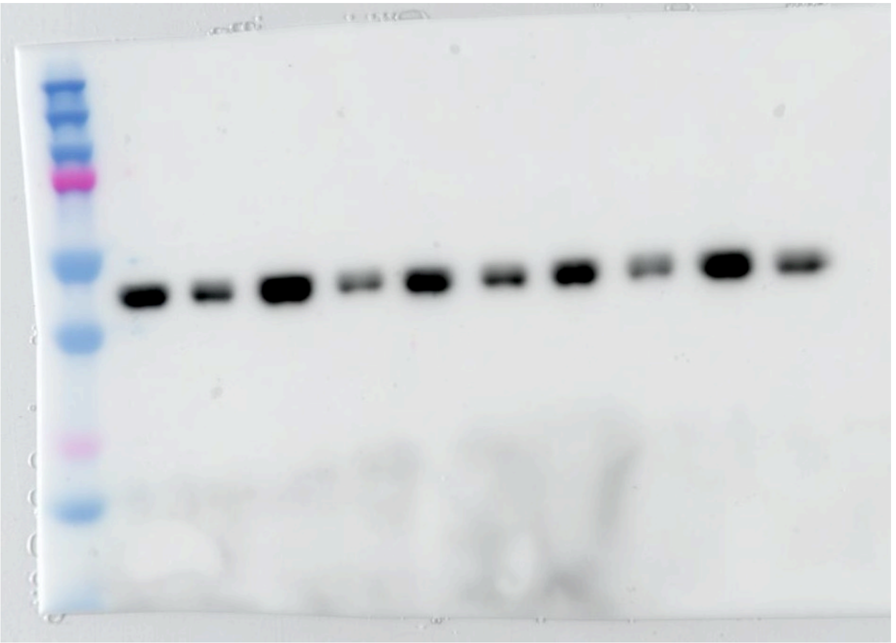

Figure 3E

P-ERK

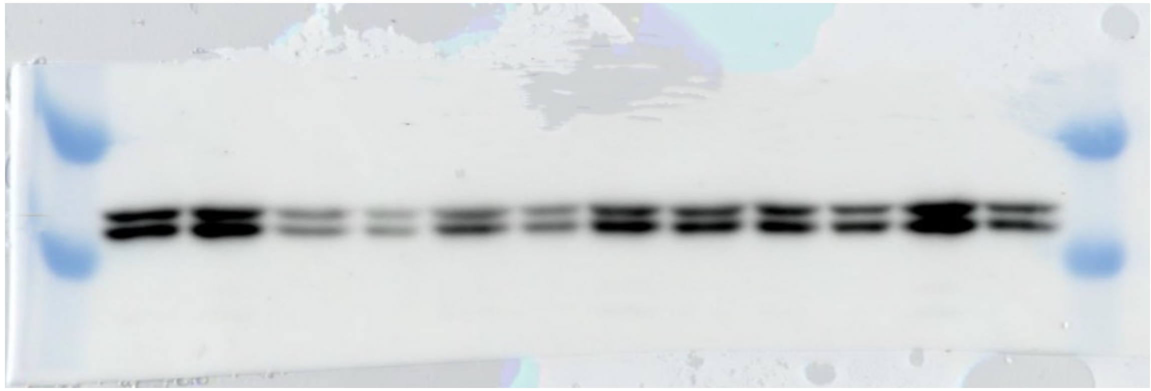

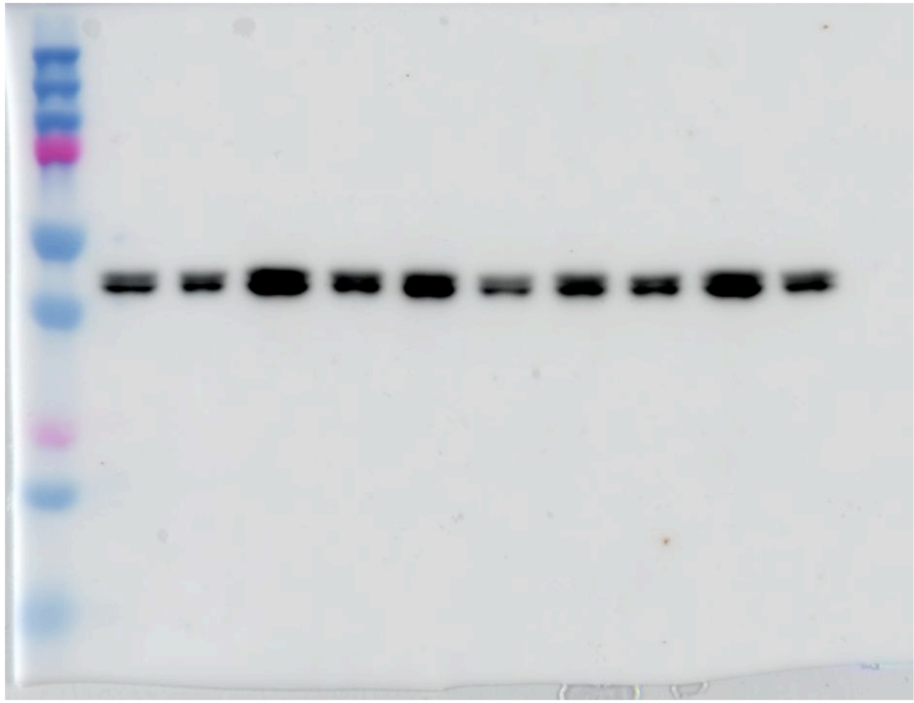

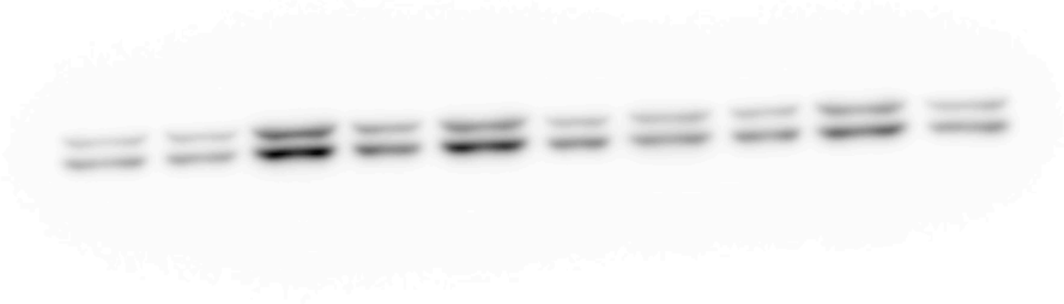

Figure 3E

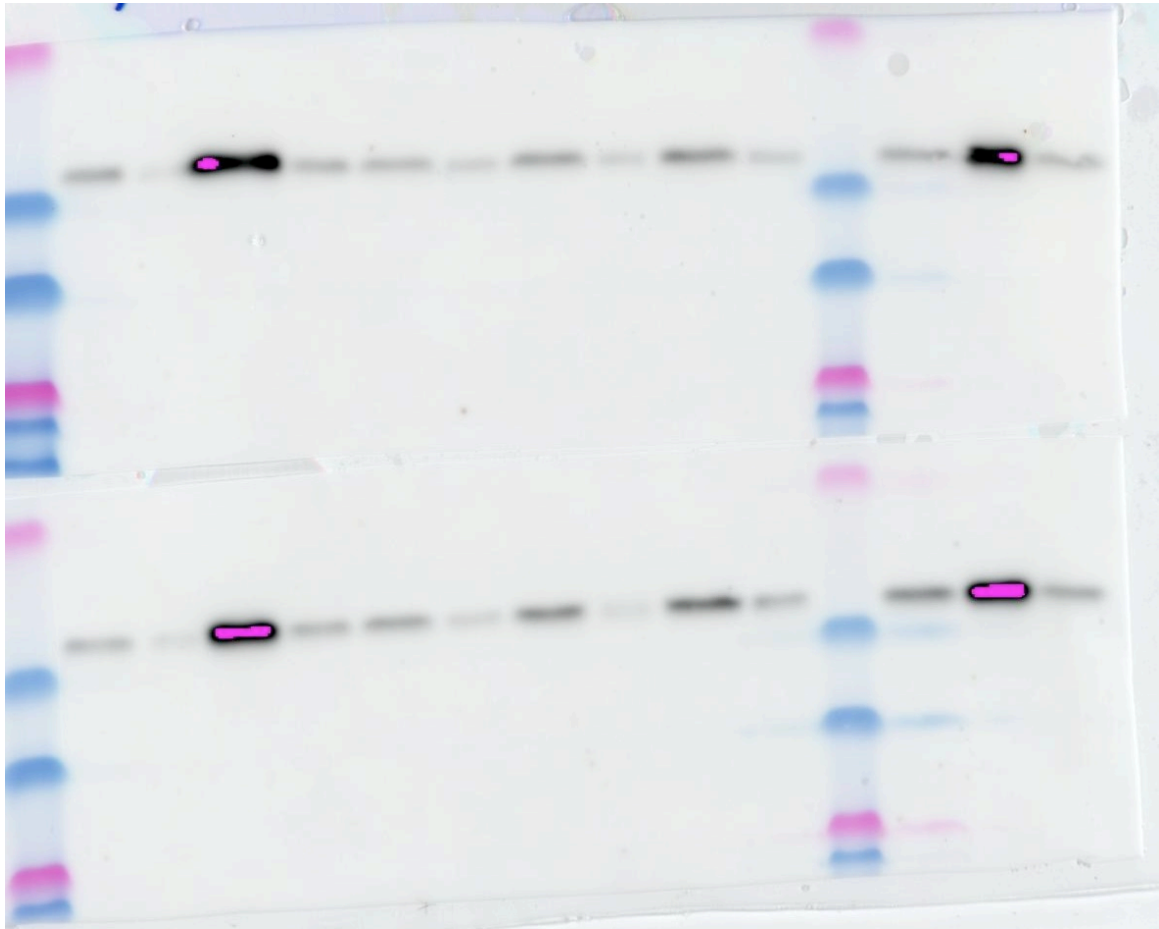

Cyclin D1

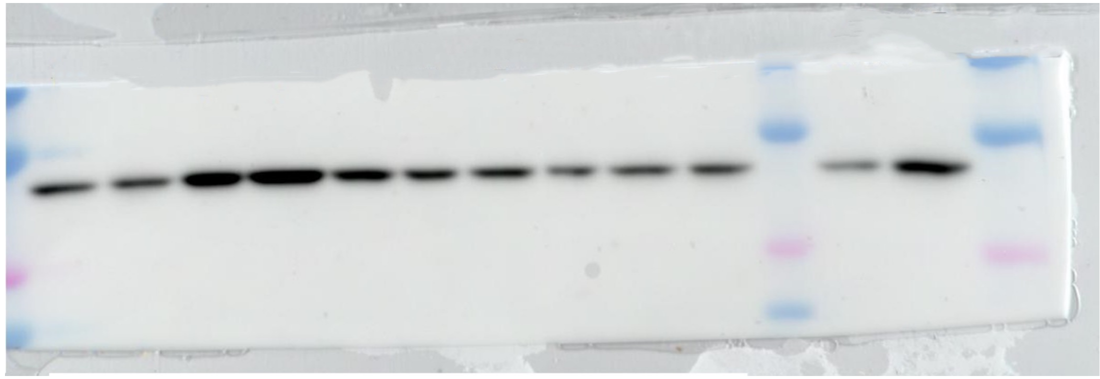

Figure 3E

C-MYC

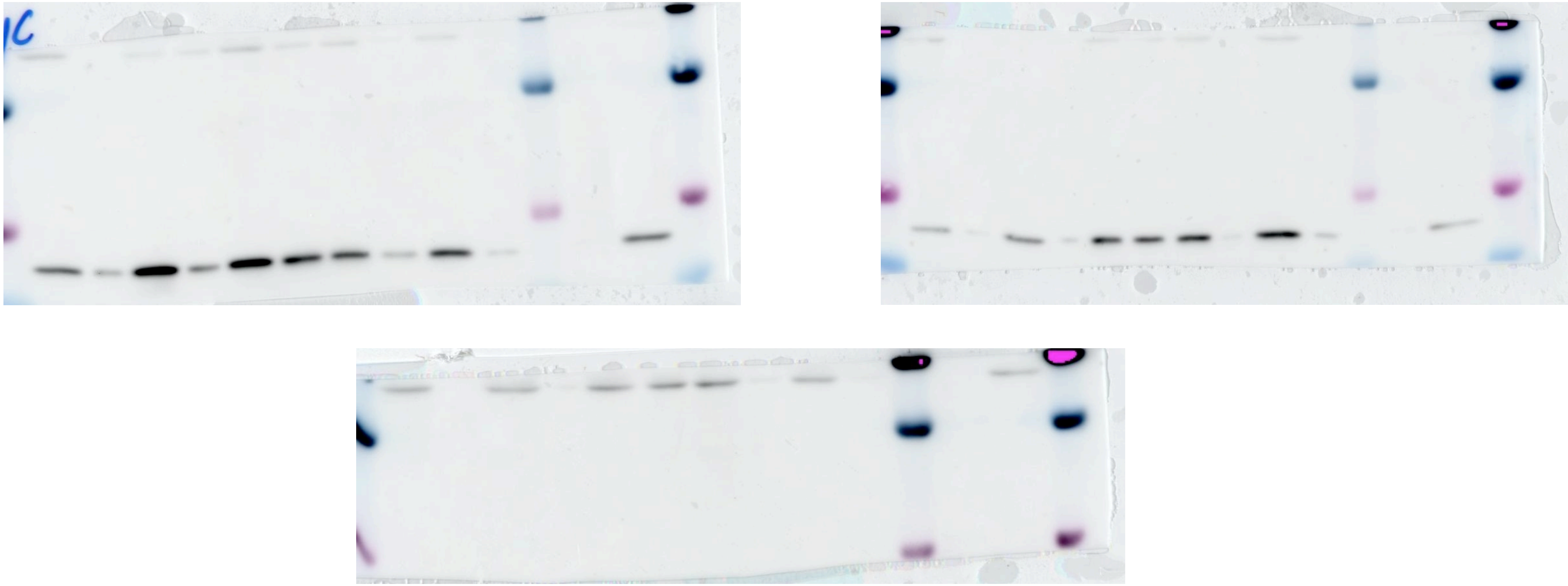

Figure 3E

GAPDH

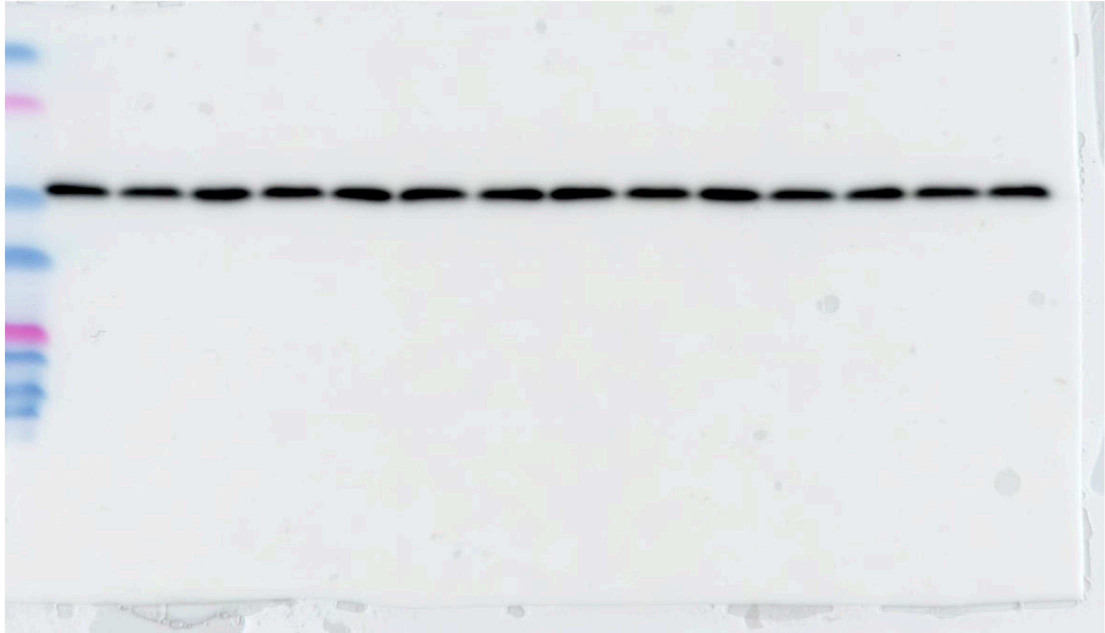

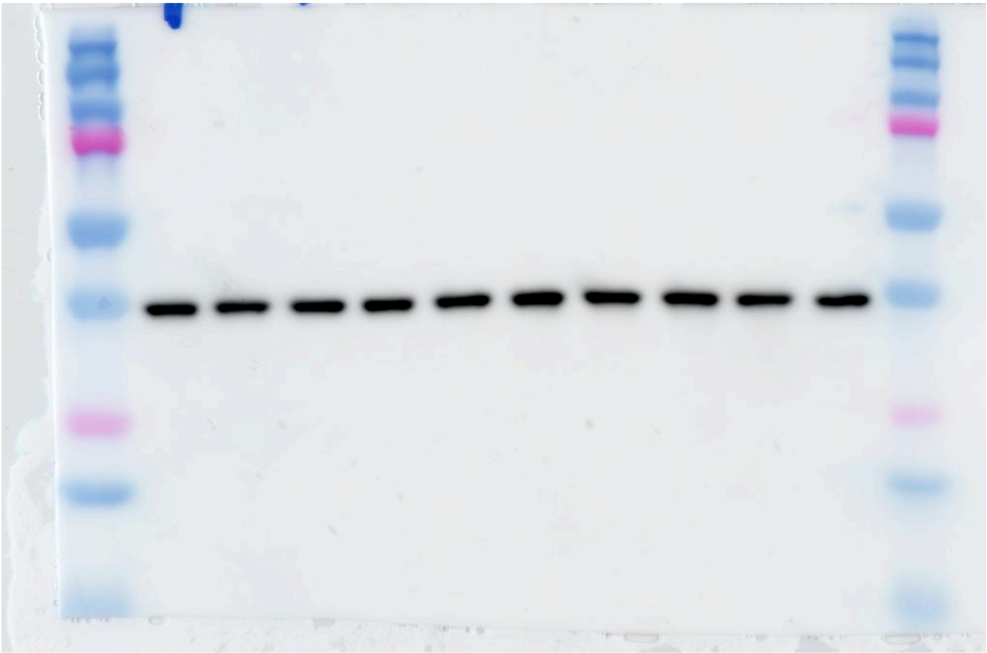

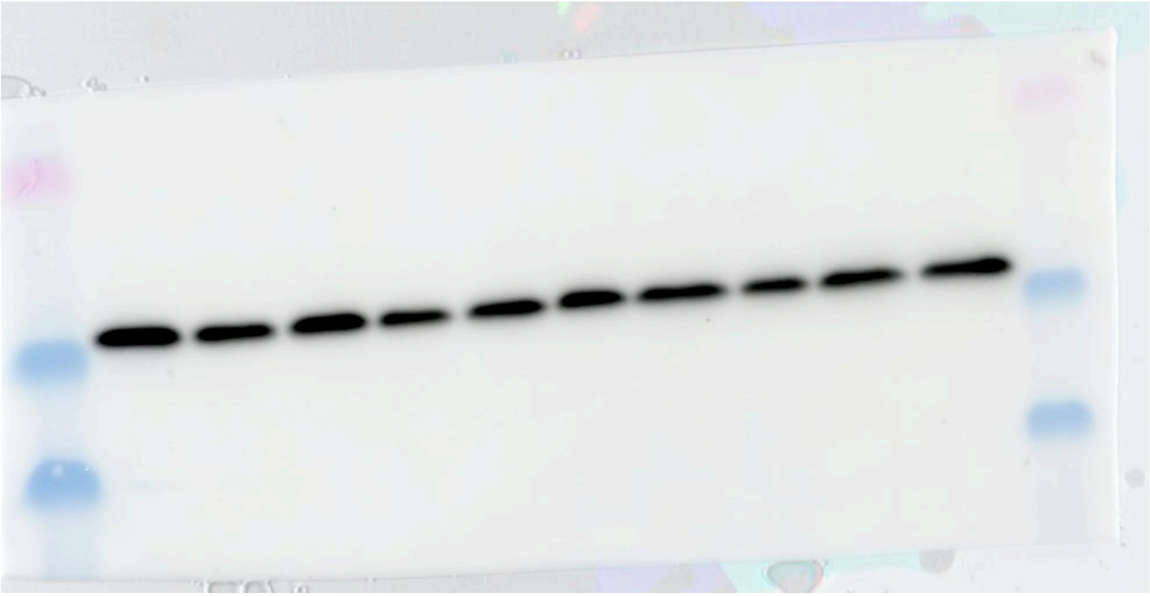

Figure 3J

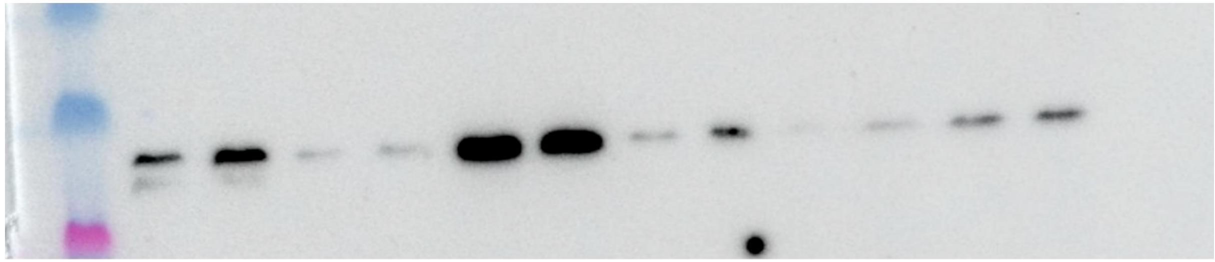

C-MYB

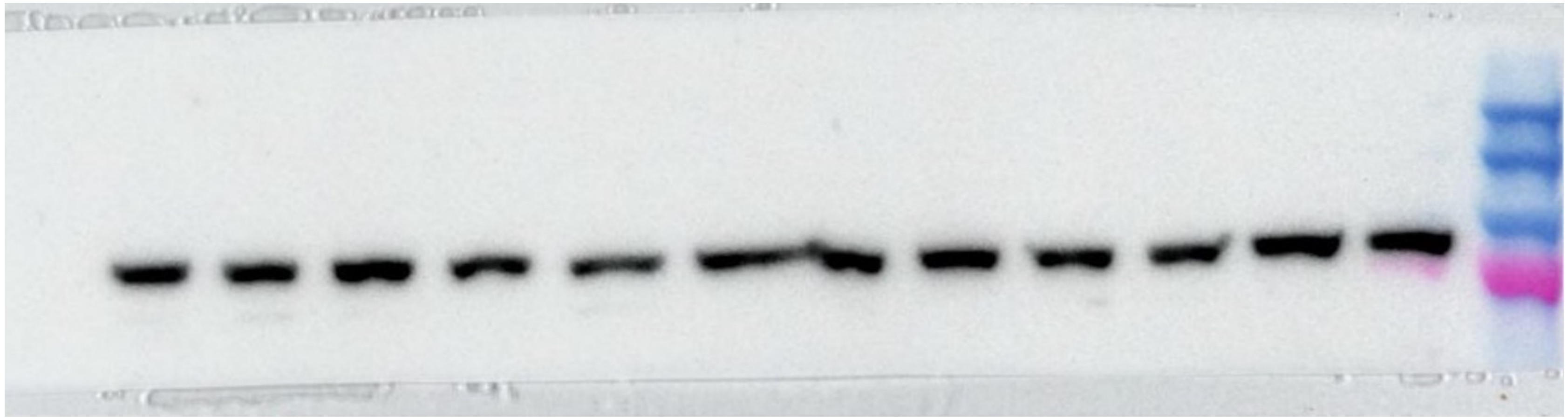

Figure 3J

P-MEK

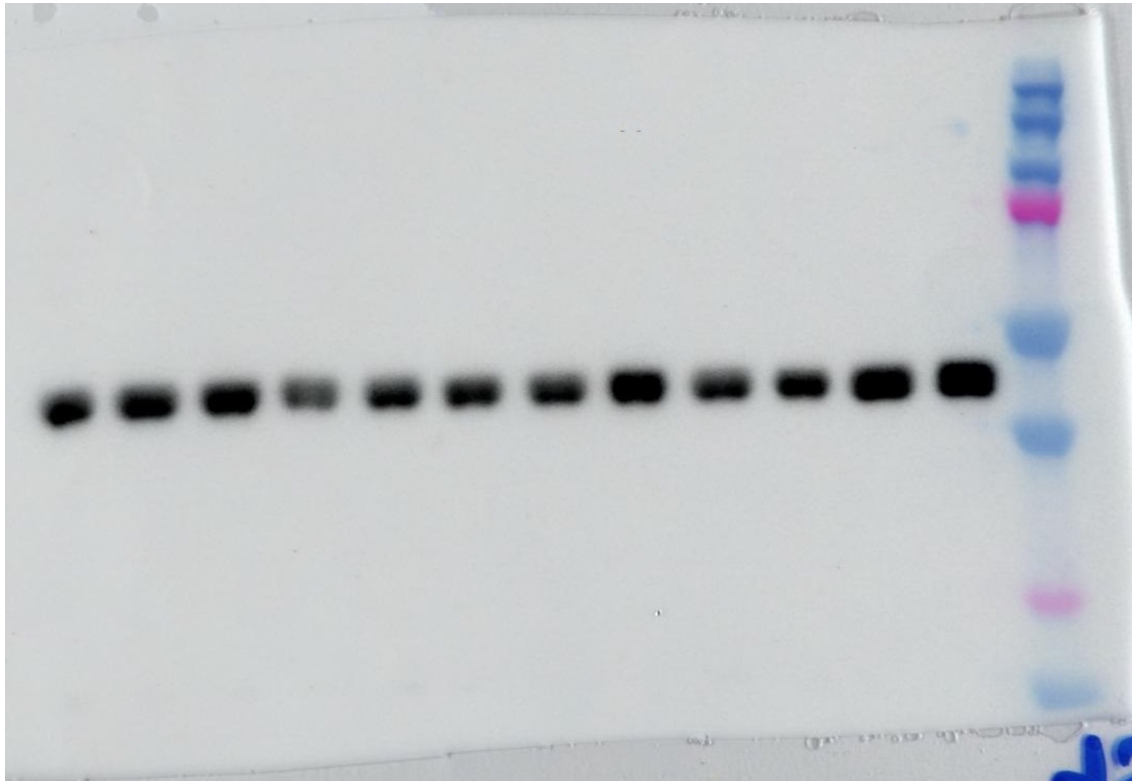

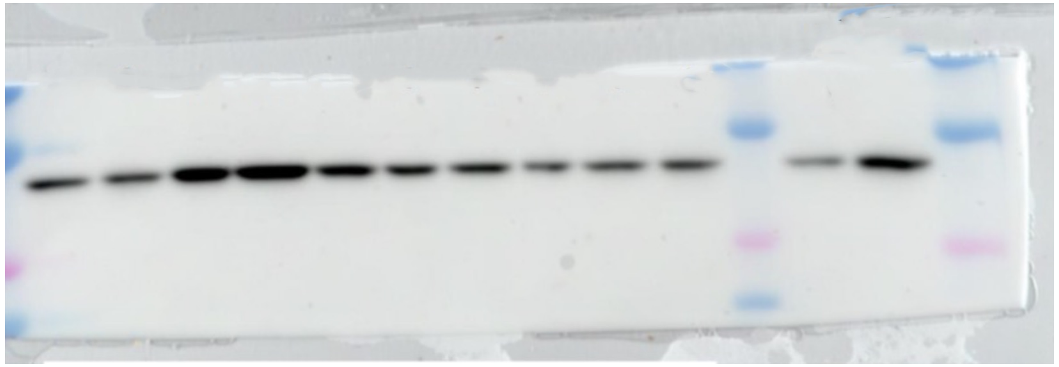

Cyclin D1

P-ERK

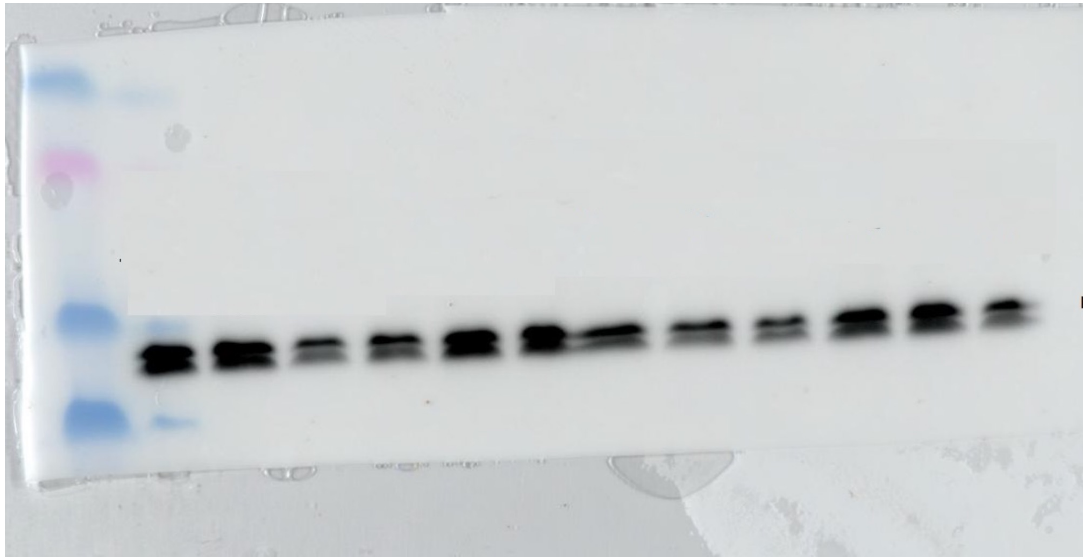

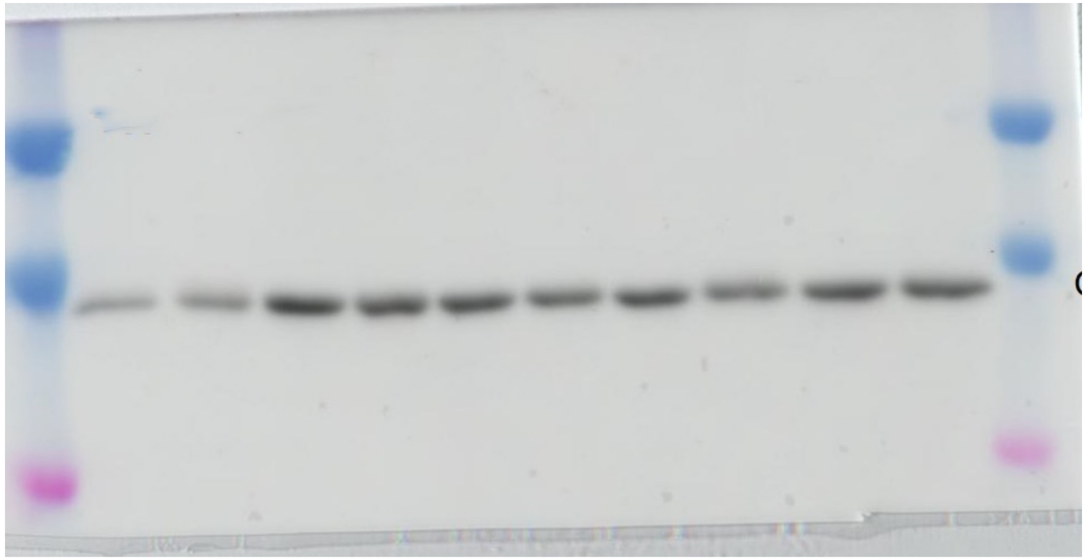

Figure 3J

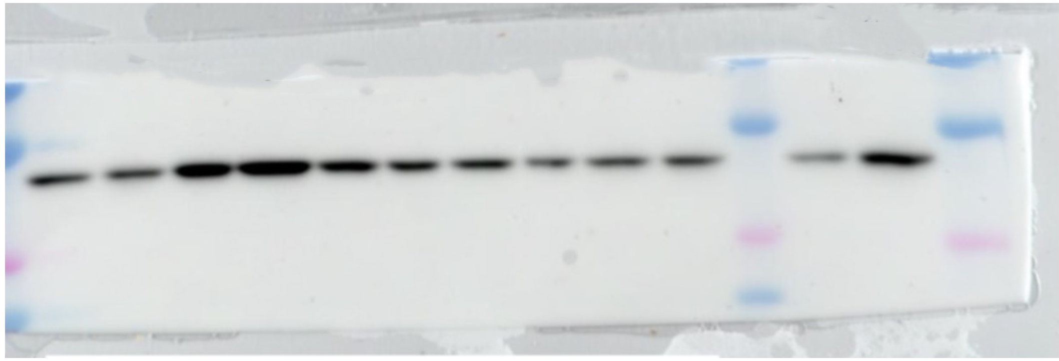

Cyclin D1

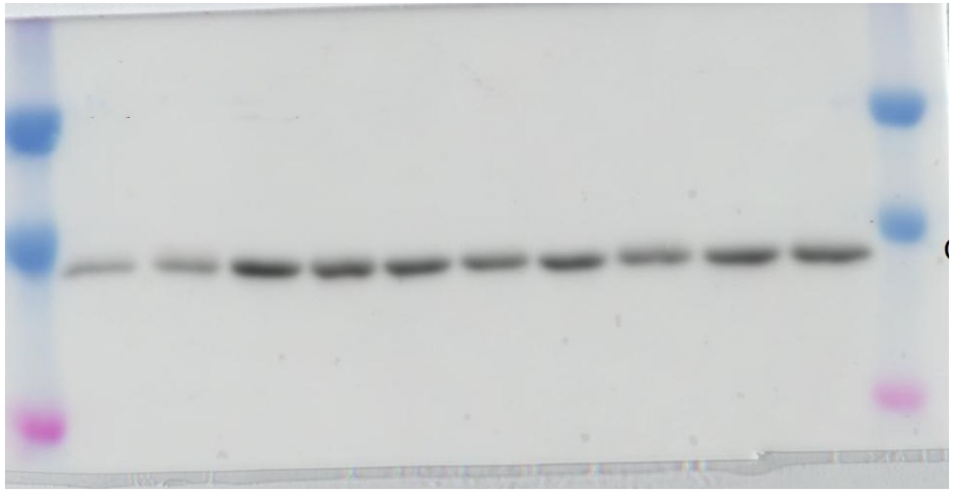

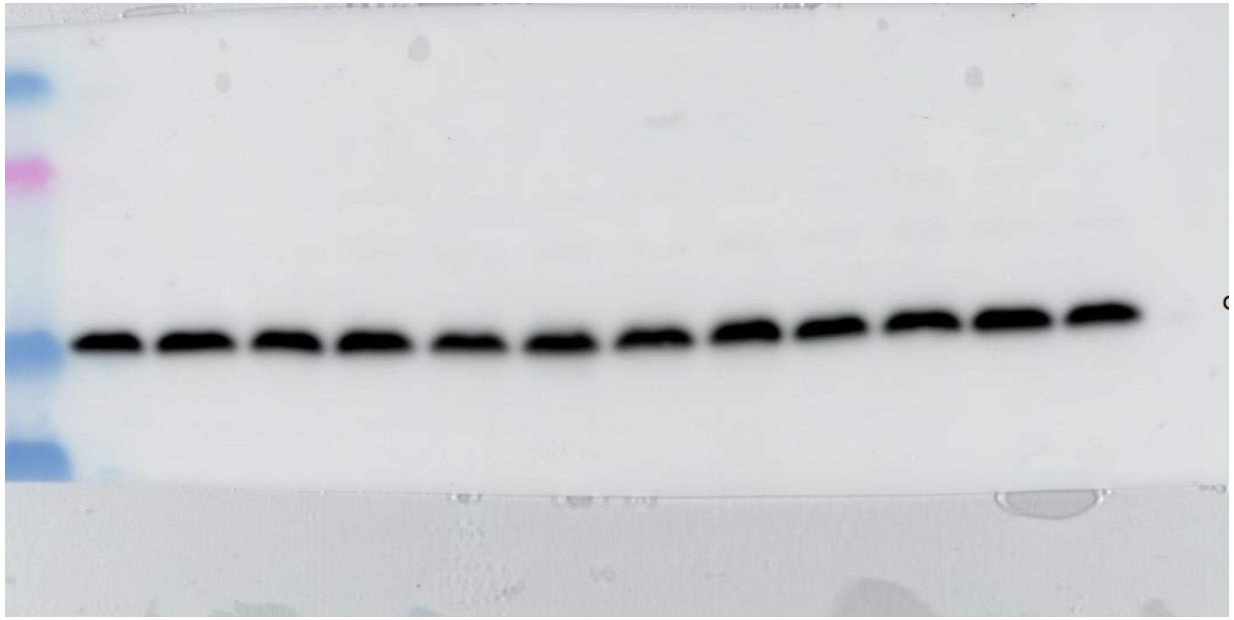

Supplement: Supplementary file 2 — Original Data File [file 41420_2023_1364_MOESM2_ESM.pdf]
